# Recent advances in multidimensional ultrafast spectroscopy

**DOI:** 10.1098/rsos.171425

**Published:** 2018-01-31

**Authors:** Thomas A. A. Oliver

**Affiliations:** School of Chemistry, Cantock's Close, University of Bristol, Bristol BS8 1TS, UK

**Keywords:** ultrafast spectroscopy, multidimensional optical spectroscopy, two-dimensional electronic–vibrational spectroscopy, pulse shaping

## Abstract

Multidimensional ultrafast spectroscopies are one of the premier tools to investigate condensed phase dynamics of biological, chemical and functional nanomaterial systems. As they reach maturity, the variety of frequency domains that can be explored has vastly increased, with experimental techniques capable of correlating excitation and emission frequencies from the terahertz through to the ultraviolet. Some of the most recent innovations also include extreme cross-peak spectroscopies that directly correlate the dynamics of electronic and vibrational states. This review article summarizes the key technological advances that have permitted these recent advances, and the insights gained from new multidimensional spectroscopic probes.

## 1. Introduction

Multidimensional optical spectroscopies have unravelled a wealth of structural, energetic and dynamical information about molecular, biological and nanomaterial systems. These studies have been able to probe phenomena spanning: quantum coherence in natural light harvesting, [1–7], exciton dissociation in photovoltaic thin films, [8–11] bound exciton pair correlations in quantum wells and vibrational dynamics in solid-state materials [12–16]. They have been decisive in determining specific molecular motions involved in protein folding [17–19] and structural dynamics [20–23], as well as revealing mechanistic insights into complex non-radiative relaxation [24–27] and chemical reaction or solvation dynamics [24,28–38]. The key advantage that two-dimensional (2D) ultrafast spectroscopies offer over one-dimensional counterparts, such as transient absorption or transient grating, is the correlation between broadband excitation and emission frequencies as a function of system evolution, which enables the resolution of homogeneous (anti-diagonal) and inhomogeneous (diagonal) line shape components. Changes in the 2D line shapes report on the frequency–frequency correlation function (FFCF). The dynamics of the FFCF yield information about the amplitudes and timescales associated with changes in the optical frequencies investigated, caused by changes in molecular structure, or evolution of the surrounding solvent structure or protein [29,31,32,39].

The additional information content available in these multidimensional experiments allows, in most cases, for the simultaneous resolution and detection of overlapping ground state bleach/stimulated emission and excited state absorption features.

**Figure 1.**
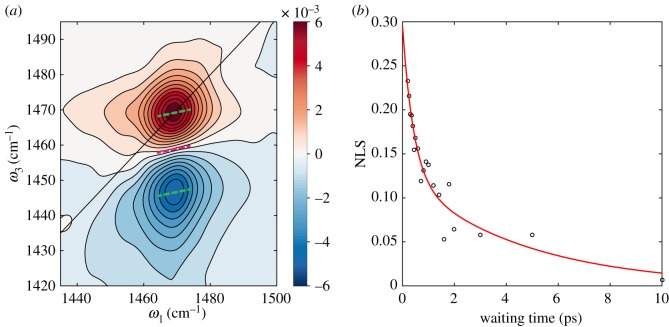
(*a*) Isotropic absorptive total 2DIR spectrum of methyl ammonium lead iodide perovskite thin film for waiting time 250 fs. The black line indicates the diagonal of the 2D correlation spectrum. The dashed green lines show fits to the centre line slopes (CLS) for the ground and excited state vibrational features. The pink line is a fit to the nodal line slope (NLS). (*b*) Time-dependent gradient of the NLS extracted from 2DIR data (open circles) and bi-exponential fit to data (red line).

Figure 1*a* shows an example two-dimensional infrared (2DIR) spectrum that illustrates these features. The 2DIR spectrum was acquired for a methyl ammonium (CH_3_NH_3_^+^) lead iodide perovskite (MAPbI_3_) thin film sample. The mid-infrared pump and probe lasers are resonant with the ${\text{NH}}_{3}^{+}$ symmetric stretching vibration of the $\text{C}{\text{H}}_{3}{\text{NH}}_{3}^{+}$ moiety and acquired for waiting time = 250 fs. The 2D correlation spectra (displayed as changes in transmission) contain two features which arise from two distinct third-order nonlinear response pathways; the positive peak corresponds to ground state emission/stimulated emission between the 0–1 vibrational states of the ${\text{NH}}_{3}^{+}$ symmetric stretching vibration. The negative feature corresponds to an excited state absorption as probed via the 1–2 overtone. In this early waiting time 2DIR spectrum, both features are slightly elongated along the diagonal, as illustrated by fits to the centre line slopes (CLSs, green lines) and nodal line slope (NLS, pink line). The NLS and CLS are incisive probes of changes in the local environment experienced by functional groups in 2DIR, and/or a reflection of any system-bath coupling. For increased values of the waiting time, correlation between initial excitation (*ω*_1_) and eventual emission (*ω*_3_) frequencies is lost, as reflected by the decay in the NLS, figure 1*b*. The decay in NLS was fit to bi-exponential decay, yielding time constants of 430 fs and 4.6 ps, similar to those previously determined periods for wobbling/tumbling motions of the methyl ammonium cation inside the lead iodide cage from 2DIR anisotropy measurements and molecular dynamics simulations [16]. Such re-orientation means that the ${\text{NH}}_{3}^{+}$ symmetric stretching mode will experience a variety of slightly different electrostatic potential associated with different faces and/or edges of the PbI_3_ lattice. In turn, this leads to slight shifts in the vibrational potential and associated transition frequencies, resulting in the observed spectral diffusion.

**Figure 2.**
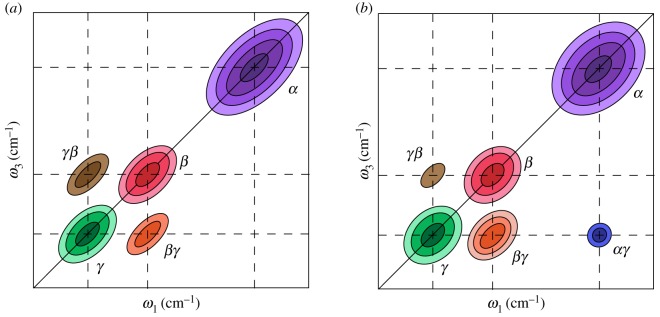
Illustrative 2D electronic spectra for a multi-chromophore system, comprised from excitons *α*, *β* and *γ*, for *t*_2_ = (*a*) 0 ps, and (*b*) >>0 ps. For simplicity only ground state bleach signals are depicted.

2D ultrafast spectroscopies can also resolve cross-peaks that appear at different absorption and emission wavelengths. The time-dependent amplitudes of cross-peaks can be crucial to determine the associated timescale for chemical exchange, structural changes or anharmonic coupling via 2DIR spectroscopy, or to follow the flow of energy between different excitons in a coupled multi-chromophore system using 2D electronic spectroscopy (2DES). A schematic 2DES spectrum for the latter scenario is illustrated in figure 2 for a system containing three excitons (bound electron-hole pairs), *α*, *β*, *γ*. For the sake of simplicity, peaks are only displayed for ground state bleach pathways. The presence and time-dependence of cross-peaks between the diagonal features reveals physical insights into the nature of energy transfer between the three excitons. For the time zero 2DES spectrum in figure 2*a*, intense cross-peaks between diagonal features *γ* and *β*, at *βγ* and *γβ*, reveal that the two excitons are strongly coupled, displaying ‘up-’ and ‘down-’ hill energy transfer pathways. At later waiting times (figure 2*b*), spectral diffusion results in a loss of elongation along the diagonal for most features, and the intensity of the *βγ* cross-peak increases relative to that of *γβ*, as population relaxation starts to dominate over coherent energy transfer. A weak intensity cross-peak, *αγ*, rises on a far longer timescale and is not accompanied by a corresponding cross-peak at *γα*, indicating that excitons *α* and *γ* are weakly coupled, and that energy transfer occurs via population relaxation, i.e. incoherently. No cross-peaks are present between *α* and *β*, informing us that the two states are not dipole coupled.

Initial 2D ultrafast spectroscopy experiments were confined to very specific parts of the electromagnetic spectrum, such as the mid-infrared [40–43] and visible [24,44,45]. Historically, this is in part because such pioneering experiments emerged from forerunner narrowband three-pulse photon echo spectroscopies at similar frequencies [46–51], and were driven by the emergence of broadband optical parametric amplifiers (OPA) with commensurate short pulse durations across the visible and in the mid-infrared [52–55]. It was also relatively straightforward to create the necessary phase-locked pulse pairs across these wavelength regions compared with, for example, ultraviolet light.

The advent of pulse-shaping technology had a significant impact on the multidimensional optical spectroscopy field, in tandem with advances in ultrafast laser technology such as the increased availability of high-power, short-pulse, regenerative amplifiers with repetition rates up to 100 kHz. These developments led to a wider variety of broadband laser sources and the ability to vary time delays between pulses with attosecond precision, or fully compress pulses to the limit of their time-bandwidth product. The consequence for this community is that 2D ultrafast correlation spectroscopies are now routine throughout terahertz (THz), infrared, visible and ultraviolet parts of the electromagnetic spectrum.

Many aspects of 2D optical spectroscopies have been detailed by several comprehensive reviews since their inception [56–69]. In this review, I highlight some of the recent advances in the field such as extreme cross-peak spectroscopies, 2D action spectroscopies such as 2D fluorescence or photocurrent phase modulated spectroscopies and surface-specific 2DIR via sum-frequency generation (SFG) detection. Further, the details of new 2D Raman spectroscopies, as well as 2D spectroscopies that reach into the THz domain exploring low-frequency and phonon modes, or fundamental properties of materials, are discussed. I conclude with some future trajectories for the field.

## 2. Key technological developments

Several of the advances in multidimensional ultrafast spectroscopies highlighted in this article have only been realized due to the major advances in femtosecond pulse generation and the amplitude and phase control of laser light with pulse shapers such as acousto-optic modulators (AOMs) or spatial light modulators (SLMs). Femtosecond pulse-shaping technology has been widely adapted by the community, making them indispensable pieces of equipment for either dispersion management, phase cycling and/or multiple pulse generation. In this section, I provide a very brief precis of how 2D spectroscopy experimental approaches have evolved since their inception, and the current state of the art.

**Figure 3.**
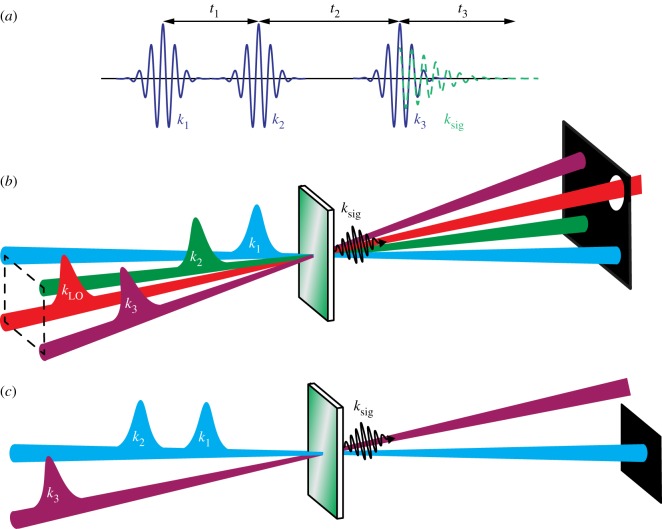
(*a*) Pulse sequence for 2D optical spectroscopies, (*b*) ‘boxcar’ geometry and (*c*) pump–probe geometry.

From the outset, it is important to define nomenclature that will be used throughout this review for the various *k*-vectors associated with pulses and the inter-pulse time delays. The third-order nonlinear spectroscopic pulse sequence, *k*-vectors and time delays defined are in figure 3*a*, and will be described in the context of a mixed time-frequency acquisition scheme which has become the de facto method for 2D ultrafast spectroscopies. For a model three-level system with ground state |*g*〉, first and second excited states |*e*〉 and |*f*〉, an ultrashort broadband laser pulse, *k*_1_ creates coherences between |*g*〉 and |*e*〉 states. The system then evolves for a specific coherence time, *t*_1_, before a second pump photon, *k*_2_, converts the system into a population of either |*g*〉 or |*e*〉 states. The system is then allowed to evolve on the ground or excited states for a fixed value of the waiting time, *t*_2_, (or pump–probe time delay) before interacting with the probe pulse, *k*_3_. The probe pulse drives the system into a second coherence, either between |*g*〉 and |*e*〉 or |*e*〉 and |*f*〉 states, before emitting the signal, *k*_sig_, at echo time *t*_3_. Typically, the signal pulse is frequency dispersed onto a multi-element array detector, transforming the time-domain signal into its conjugate, *ω*_3_. This is repeated for many values of *t*_1_ to generate *t*_1_ − *ω*_3_ 2D maps. The *t*_1_ coherence time must be sampled in sufficiently small *δt*_1_ steps to properly sample the Nyquist period of oscillations associated with coherences between |*g*〉 and |*e*〉 states. The data are subsequently windowed, apodized and Fourier-transformed along the *t*_1_ axis to generate a 2D *ω*_1_–*ω*_3_ correlation map, for a specific value of the waiting time. Depending on the vertical Franck–Condon factors between the |*g*〉 and |*e*〉 states, and the laser bandwidth used, electronic, vibronic or vibrational coherences will be launched and propagated, leading to oscillations in the corresponding 2D spectra as a function of the waiting time [1,3,9,23,70–73].

Mixed time-frequency acquisition schemes for 2D spectroscopy, as described above, are now almost universally favoured over wholly frequency domain (e.g. hole-burning) due to the distortions in 2D line shapes the latter scheme can introduce. It also helps to maintain high temporal resolution. For a direct comparison, see [74].

### 2.1. Beam geometries and pulse shaping

One of the most popular experimental configurations for acquisition of 2DES and 2DIR spectra is in the fully non-collinear ‘boxcar’ geometry (shown in figure 3*b*), which has distinct advantages over the partially collinear (pump–probe) geometry (figure 3*c*): (i) the emitted signal field is background free, and heterodyned by a local oscillator (LO) to extract the phase and amplitude information of the third-order nonlinear response. The background-free nature of the measurement is estimated to increase the signal-to-noise ratio compared with a pump–probe geometry by up to a factor of 19.5; [75] (ii) the rephasing and non-rephasing signals are collected separately, but are emitted in the same phase-matched direction [24]. The total 2D frequency-resolved photon echo signal is the sum of these two different measurements; (iii) because the two pump pulses are independent laser beams, the polarization of *k*_1_ and *k*_2_ can be independently controlled and thereby used to implement cross-polarized pump-pair pulse sequences that can greatly enhance the intensities of cross-peaks over diagonal features [76–79].

With these advantages come associated experimental difficulties, such as maintaining phase stability between the pump pulse pair which hampered early 2DES experimental efforts [44]. This problem was initially circumvented using a passive-phase stabilization approach, where *k*_1_ and *k*_2_ are generated using a diffractive optic, and the *t*_1_ time delay controlled by translating wedge pairs [24,80]. Methods in which the *t*_1_ time delay is actively stabilized have also been successfully demonstrated [81–83]. Alternatively, beamsplitters have been used to generate pulse pairs, and the time delay controlled with piezo-electric delay stages with sub-nanometre linear resolution. The latter method has the significant advantage that the time delay is created by using reflective optics, and additional chirp is not introduced to the broadband laser pulses. The principal problem with acquisition in the boxcar geometry is that the signal is emitted non-collinear to the probe beam, and the phase relationship between *k*_3_ and *k*_sig_ must be established by additional measurements, such as pump–probe, under the same experimental conditions [24,84]. If the phase relationship is not properly established, the real and imaginary components can mix, leading to dispersive distortions in the absorptive 2D correlation spectra, as highlighted in [85].

The background-free approaches to 2D spectroscopies described thus far, require the physical translation of delay stages to create the *t*_1_ time axes in mixed time-frequency 2D measurements. Several groups have demonstrated an alternative approach, which allows an entire 2D spectrum to be recorded in a single laser shot [86–88]. The 2DES variant, gradient-assisted photon echo spectroscopy (GRAPES), combines the background-free geometry, and inter-pulse time delays are controlled by piezo-electric stages, but pulses are cylindrically focused to a vertical line in the sample. Tilting the pulse front of *k*_1_ relative to *k*_2_ spatially encodes the *t*_1_ time delay in the vertical dimension. The resulting signal is spatially imaged into a spectrograph to record an entire *t*_1_–*ω*_3_ spectrum every shot. This significantly decreases the acquisition time for 2D spectroscopy, making it comparable to pump–probe spectroscopy. There is one pitfall, however; samples must be spatially homogeneous (over a length scale of several millimetres), which partially restricts measurements from investigating dynamics of systems in low-temperature glasses which are generally inhomogeneous.

The partially collinear, or pump–probe, geometry (figure 3*b*) removes the problem of phase matching in experiments where very different pump and probe frequencies are used [89], and greatly reduces the complexity of the alignment procedures and experimental design, as only two beam paths are required. 2D signals in the partially collinear geometry are emitted collinearly with *k*_3_ (*k*_s_ = ± *k*_1_ ∓ *k*_2_ + *k*_3_), which acts so as to ‘self-heterodyne’ the signal, with the consequence that the *k*_3_–*k*_sig_ phase relationship is known, and 2D spectra are automatically phased [74,90,91]. Consequentially, however, the rephasing (*k*_s_ = –*k*_1_ + *k*_2_ + *k*_3_) and non-rephasing (*k*_s_ = +*k*_1_ − *k*_2_ + *k*_3_) signals are detected simultaneously and cannot be separated, which may hamper the isolation of specific spectroscopic pathways [92,93]. In the mid-IR, the *t*_1_ delay is often controlled by a Michelson interferometer. The shorter wavelengths associated with visible, and especially ultraviolet, radiation mean that phase-stability issues are more problematic using such an approach. Such problems have been circumvented in the visible domain with wedge-based systems such as translating wedge-based identical pulses encoding system (TWINS) [94,95]. In all cases, to maintain phase relationship between *k*_1_ and *k*_2_ still requires monitoring or post-processing to correct for timing errors, and pump-scatter is a far greater problem in this geometry. Further, the 2D signal of interest is emitted collinearly with the pump–probe signal. The 2D signal oscillates in *t*_1_, whereas the pump–probe signal decays, and thus Fourier-transform along the *t*_1_ axes removes the pump–probe background.

The disadvantages of the partially collinear geometry were surmounted with the introduction of pulse-shaping technology such as AOMs or SLMs [91,96–98] to multidimensional optical spectroscopy. These devices were used to generate phase-locked pulse pairs and control of the relative phases [74,91,96,99]. The groups of Zanni and Olgivie pioneered these approaches for 2DIR and 2DES, respectively, demonstrating that the pump–probe background could be completely removed by ‘phase cycling’ *k*_1_ and *k*_2_ pulses. By changing relative phase between *k*_1_ and *k*_2_ (*ϕ*_12_), the phase of the emitted 2D signal is correspondingly altered, but as the pump–probe signal is insensitive to the phase modulation, and through judicious choice of several values of *ϕ*_12_ and appropriate summation of the resulting signals, the pump–probe background can be removed. This method can also be used to isolate the rephasing and non-rephasing signals in the partially collinear geometry [91,96,100]. Pulse shapers also offer other significant advantages to physically scanning the *t*_1_ delay: (i) With acousto-optic pulse shapers, the pump pulse pair can be refreshed at the repetition rate of up to several kHz, matching the output of many regenerative amplifiers. (ii) Phase cycling does not reduce the duty cycle of experiments. (iii) A pulse shaper allows 2D spectra to be collected in the fully rotating frame, where *ϕ*_12_ is incremented with the *t*_1_ time delay to shift the fundamental frequency to zero. Resultantly the signal will not oscillate as a function *t*_1_, and the envelope of the signal is detected, rather than its fundamental frequency. The rotating frame thus provides a convenient way to under-sample *t*_1_, but in practice, many experiments use a partially rotating frame, where the fundamental frequency is not shifted all the way to zero, but to a frequency with an associated longer Nyquist period. The other significant advantage of using a rotating frame is that pump-scatter can be shifted away from the desired signals [74]. (iv) Compressed sensing is routinely used in 2D NMR spectroscopy, and requires only a fraction of the usual number of measurements to retrieve the same spectrum recorded if *t*_1_ were fully sampled. The same principles apply to 2D ultrafast spectroscopies, the *t*_1_ delay can be randomly sampled with a normal distribution around *t*_2_ = 0 fs, but with uneven *δt*_1_ steps. With no *a priori* knowledge of the frequencies of interest that should be sampled in *t*_1_, the data can be recovered using complex algorithms. Such schemes are only practical if the *t*_1_ time delay can be changed from shot to shot, and using a partially rotating frame, i.e. with a pulse shaper. A study that applied compressed sensing to 2DIR spectroscopy, reported a 4× speed-up in the acquisition time compared to conventionally sampled 2DIR data [101,102].

As outlined here, there are advantages and disadvantages to the ‘boxcar’ and partially collinear geometries using pulse shapers. Hybrid methods have been used to combine the advantages of the background-free ‘boxcar’ geometry and pulse shapers to generate inter-pulse time delays and *ϕ*_12_ [75,103].

### 2.2. Generation of ultrashort pulses from terahertz to ultraviolet

The rapid commercialization of chirped pulse amplifiers has led to the widescale availability of low-noise high-power laser sources, which in turn has driven further innovations in tunable ultrashort laser sources for 2D spectroscopy, that now span wide portions of the electromagnetic spectrum. There are many approaches to generating these desired sources of light, which are briefly overviewed here. Broadband visible light sources for 2D spectroscopies are generated via two main methods: non-collinear optical parametric amplification (NOPA) [54,55,104], or filamentation of 800 nm in rare gas mixtures (e.g. [105]). The latter has been implemented in conventional ‘boxcar’ or GRAPES background-free configurations of 2DES [106–108]. Recently white light generated from supercontinuum generation in sapphire has been used in combination with a pulse shaper for 2DES measurements of carbon nanotubes [109]. Generation of ultrashort sub-40 fs ultraviolet pulses poses greater challenges. Four-wave mixing in a hollow-core fibre of 800 and 400 nm in rare gases has generated less than 35 fs pulses centred between 266 and 275 nm [110,111], taking advantage of the readily available harmonics of the Ti : sapphire fundamental of the femtosecond amplifiers. More tuneable UV-pulses have been generated by either SFG of broadband NOPA outputs with narrowband higher power 800 nm laser pulses [112], or achromatic doubling using cylindrical focusing to satisfy a broader range of phase matching conditions [113]. Near transform-limited octave-spanning mid-infrared sources have emerged broadly using similar techniques such as filamentation in rare-gas mixtures [114,115], and use deformable mirrors to correct for nonlinearities in the spectral phase. The dawn of sub-picosecond THz pulses with sufficient field intensity to drive nonlinear spectroscopy measurements, meant that multidimensional ultrafast experiments involving THz radiation have finally come of age. THz sources for nonlinear spectroscopic measurements can be generated in several ways; collinear phase matching in nonlinear crystals such as ZnTe with intense 800 nm field; 800 and 400 nm dual pumping of rare gas or air to drive plasma generation or non-collinear phase matching with tilted 800 nm pulse fronts in LiNbO_3_ crystals [116,117].

## 3. Extreme cross-peak multidimensional spectroscopy

Typically 2D ultrafast spectroscopies have probed the same type of transitions (e.g. vibrational or electronic), even if different central pump and probe wavelengths were used [91,118]. This changed with the advent of ‘extreme cross’ peak spectroscopies [119] such as 2D electronic–vibrational (2DEV) [120] and 2D vibrational–electronic (2DVE) [121] spectroscopies. These mixed electronic–vibrational spectroscopies are uniquely placed to investigate how the interplay and coupling between electronic and nuclear degrees of freedom dictates the non-radiative relaxation dynamics of molecules, especially those that involve conical intersections (CIs). Such dynamics underpin the functional dynamics of many systems, for example: (i) the primary steps of vision that involve *cis–trans* isomerization of rhodopsin [122,123] or (ii) the rapid excited state deactivation of DNA nucleobases [124,125].

### 3.1. Two-dimensional electronic–vibrational spectroscopy

2DEV spectroscopy can be viewed as the extreme cross-peaks between 2DES and 2DIR. To date, 2DEV spectroscopy has been used to investigate a range of photochemical dynamics of molecular systems such as the strength of solute–solvent coupling for specific high-frequency vibrations of dye molecules [126,127], and coupled electronic–nuclear motion through CIs [128]. Further, 2DEV spectroscopy has been used to investigate the time-dependent site populations of light-harvesting complex II (LHCII) [129,130].

**Figure 4.**
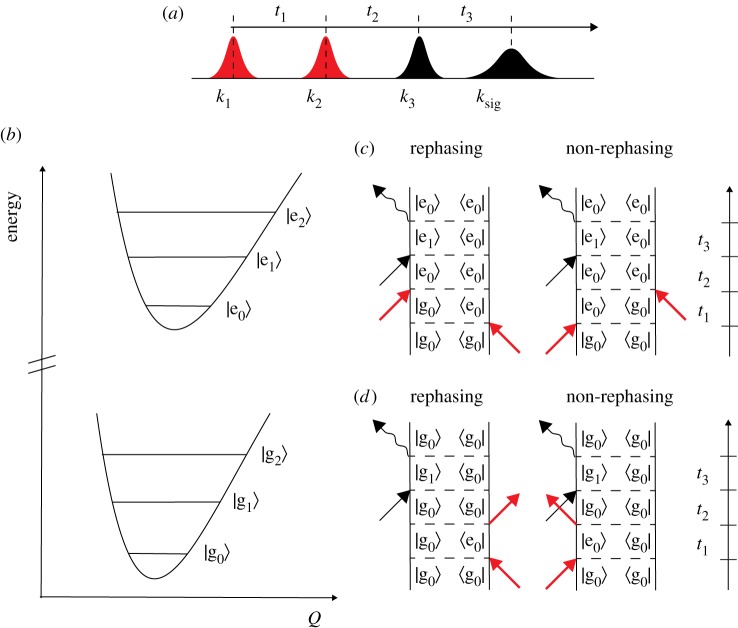
(*a*) 2DEV pulse sequence, (*b*) model system containing two electronic states with an associated vibrational manifold. Double-sided Feynman diagrams for the rephasing and non-rephasing 2DEV pathways associated with evolution during *t*_2_ on the (*c*) excited and (*d*) ground electronic states.

2DEV is part of the third-order nonlinear spectroscopy family of photon echo spectroscopies. The 2DEV pulse sequence is the same as the generic sequence described in figure 2; however, the pump and probe pulses are non-degenerate: electronic transitions are interrogated in the *t*_1_ coherence, and vibrations in the *t*_3_ period. The 2DEV pulse sequence is given in figure 4*a*. Figure 4*b* displays the energy structure for a model system containing two electronic states (*g*, *e*), each with a nestled manifold of vibrational states (0, 1, 2) as indicated by the subscripts. Figure 4*c*,*d* shows the associated double-sided Feynman diagrams that define the rephasing and non-rephasing Liouville pathways, for dynamics that propagate during the waiting time on the excited or ground electronic states, respectively. All 2D spectra shown in this review article are displayed as change in transmission (Δ*T*), and thus signals that arise from the Liouville pathways shown in figure 4*c* will be negative, whereas those from figure 4*d* are positively signed.

The signal pathways displayed in figure 4 neglect the possibility of directly exciting |*e*_1_〉 due to limited bandwidth of pump pulses. As recent theoretical simulations demonstrate, this introduces additional pathways and associated spectral features [131]. One of the notable advantages of 2DEV spectroscopy over 2DES is the limited number of Liouville pathways, meaning the assignment and interpretation of data is far less ambiguous when compared to 2DES [132,133]. In all implementations to date [120,134], 2DEV spectroscopy has been performed in the partially collinear geometry (figure 3*c*) with a pulse shaper, meaning the 2DEV spectra are automatically phased, as the mid-IR probe pulse (*k*_3_) self-heterodynes the signal (*k*_s_), with phase cycling of *k*_1_ and *k*_2_ used to remove the pump–probe background, and isolate the rephasing and non-rephasing signals if desired.

The successful implementation of 2DEV spectroscopy was first demonstrated for a model push–pull emitter dye, 4-(dicyanomethylene)-2-methyl-6-(4-dimethylaminostyryl)-4*H*-pyran (DCM) in dimethyl sulfoxide solution [120]. The 2DEV spectra of DCM revealed correlated electronic–vibrational shifts for one high-frequency vibrational mode. These observations were understood by recognizing the significant differences in geometry associated with the ground and first excited electronic state of DCM. Franck–Condon excitation places the molecules out of equilibrium on the excited state, and to reach the electronically excited state minimum, changes in the molecular geometry driven by vibrational cooling induced via solute–solvent coupling is required. To account for the shift in the observed infrared frequency, part of this reorganization must therefore involve changes in the bond lengths associated with the probed C=C/C–C backbone mode.

**Figure 5.**
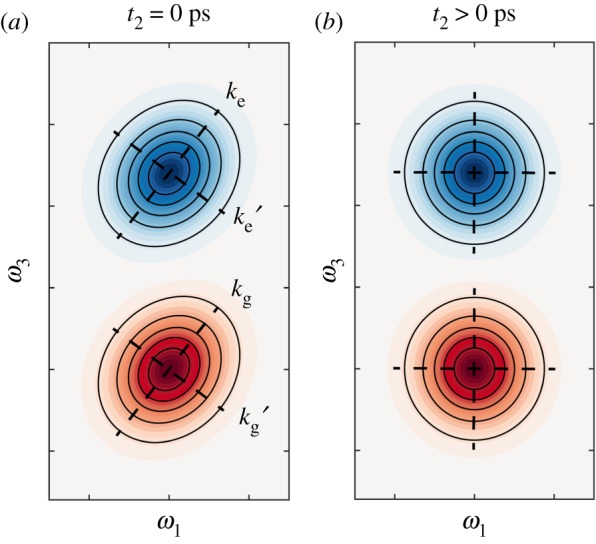
Schematic 2DEV spectrum for (*a*) early waiting times and (*b*) long waiting times. Overlaid dashed lines depict the two different CLS.

Theoretical response functions were derived to model the dynamical correlations observed between electronic and vibrational transition frequencies in 2DEV spectra for model monomeric molecules [126,127]. These studies used the energy structure shown in figure 4*b*. Independent baths were used for the electronic and vibrational degrees of freedom, given that electronic transitions will experience more rapid and intense fluctuations which are quickly damped, whereas the vibrational coordinate is fairly rigid and slowly damped. This is further justified in terms of the magnitude of the dipole moments and the respective bath spectral densities. Using Kubo line shapes and in the approximation of the impulsive limit, numerical simulations revealed that the NLS that exists between interfering positive and negative features in the total absorptive 2DEV spectra (see schematic 2DEV spectra in figure 5) decays on the same timescale as a fluctuation correlation time chosen for the probed high-frequency vibrational mode [127].

For degenerate multidimensional spectroscopic measurements, such as 2DES or 2DIR, the accumulated phase in the *t*_1_ coherence can, in principle, be removed in the *t*_3_ period via the rephasing pathway. In 2DEV spectroscopy, however, as noted above, two different types of transitions are propagated in the *t*_1_ and *t*_3_ coherences, which means that the phase accumulated in *t*_1_ cannot be entirely removed in *t*_3_.

Owing to the non-degenerate nature of 2DEV spectroscopy the diagonal and anti-diagonal CLS (as indicated in figure 5) for vibrational features on the ground or excited state (as denoted by subscript g or e) have different gradients, i.e. d*ω*_1_/d*ω*_3_ (*k*_g_ or *k*_e_) is inequivalent to d*ω*_3_/d*ω*_1_ (*k*_g_′ or *k*_e_′). This also means the limiting values for the CLS in 2DEV spectra will not be ±1, as per 2DES or 2DIR spectroscopy.

Analytical expressions for the dynamics associated with the two 2DEV CLS were directly compared with experimental data for 3,3′-diethylthiatricarbocyanine iodide (DTTCI) in deuterated chloroform [126]. Using the short-time approximation, i.e. electronic or vibrational dephasing in *t*_1_ and *t*_3_, respectively, are far shorter than the lifetime of the electronically excited state, analytical forms for the two different CLS were derived. The analytical functions showed that the 2DEV CLS are sensitive to correlated electronic–vibrational bath fluctuations, and any static inhomogeneous distribution in transition frequencies. The decay in correlation of either centre line slope for a predominantly homogeneously broadened system, such as a dye molecule in solution, was found to be dependent on the vibrational dephasing time.

The derived analytical expressions showed that the ratio of decay rates for the diagonal and anti-diagonal CLSs of a specific vibrational feature, could be used to extract the relative solute–solvent coupling strength for a vibration on the ground and excited electronic states. Thus, providing a *direct* probe of the specific spectral density associated with high-frequency vibrational modes. The only other way to extract this information using ultrafast spectroscopic investigations would be to perform 2DIR and corresponding transient-2DIR experiments.

Specifically, the decay rates for the *k*_g_ and *k*_g_′ CLS of the C=C backbone vibration of DTTCI dissolved in deuterated chloroform, revealed the coupling of a vibrational mode to the solvent bath for the 0–1 vibrational transition is stronger in the electronically excited state by a factor of 1.5 compared to the ground state. This is not surprising given that the electronically excited state has a larger permanent dipole moment compared to the ground electronic state, meaning it has a far greater polarizability and solute–solvent interactions are expected to be stronger. If vibrations that evolved in |*e*_1_〉 during *t*_2_ were observed, and the ratio of decay rates for *k*_e_ and *k*_e_′ measured, the relative coupling of |*e*_0_〉 and |*e*_1_〉 levels to the solvent bath could also be determined.

**Figure 6.**
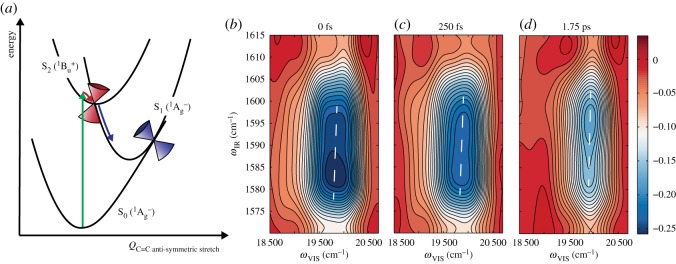
(*a*) Schematic non-radiative pathways for β-apo-carotenal in solution. (*b*–*d*) 2DEV spectra of C=C antisymmetric stretch feature of bapo in acetonitrile-*d*_3_. Adapted with permission from Oliver & Fleming [128] (Copyright © 2015 American Chemical Society).

The 2DEV spectroscopy was also used to investigate the role of CIs in the ultrafast non-radiative relaxation dynamics of a model carbonyl containing carotenoid in solution [128]. Carotenoids play a dual role in photosynthesis, acting as light-harvesting elements in parts of the solar spectrum where chlorophyll does not absorb, and as photo-regulatory elements that can help dissipate excess energy in plants under high-light intensities [135,136]. A schematic potential energy structure showing the canonical three-state model of the β-apo-carotenal (bapo) is given in figure 6*a*. The visible absorption spectrum is dominated by a strong absorption at approximately 500 nm arising from vibronic transitions to the S_2_ electronic state (^1^*B*_u_^+^). CIs are thought to play an important mechanistic role in the non-radiative relaxation from the S_2_ state, and underpin the associated short excited state lifetime (approx. 200–300 fs) [128,137,138]. CIs are formed when two (or more) potential energy surfaces intersect, and in their simplest form comprise two key dimensions: the tuning and coupling coordinates [139,140]. Motion along the tuning coordinate first brings the states into degeneracy at the point of intersection. Continued movement in this degree of freedom subsequently breaks the degeneracy. The coupling modes are formed by nuclear motions that break the degeneracy of the two states at every point along the intersection, and provide the off-diagonal coupling matrix elements to mediate surface crossing. CIs are regions of the potential energy surface where the Born–Oppenheimer approximation can break down, and electronic and nuclear motion can no longer be decoupled. In carotenoids, CIs are thought to provide a route for ultrafast deactivation to lower-lying excited states such as the $2^{1}A_{g}^{-}({\text{S}}_{1})$ state, which has eluded direct spectroscopic investigations as it is formally forbidden to one photon absorption. 2DES spectroscopy has been used to interrogate these phenomena for β-carotene [27,141] and the orange carotenoid protein [142], revealing correlations in the decay of the S_2_ → S*_n_* stimulated emission features with the onset of absorptions for S*_n_* ← S_1_ excited state absorptions. These studies, while informative, did not provide direct information relating to the specific nuclear motions that mediate the coupling between potential energy surfaces and drive passage through the conical intersection, whereas 2DEV is uniquely placed to interrogate these phenomena.

There has been some controversy surrounding the assignment of the excited state vibrational modes of bapo [143], due to lack of reliable *ab initio* calculations and an abnormally large Duschinsky rotation associated with changes in bond lengths associated with polyene backbone [128,143–145]. Fortunately, the relative orientation between the electronic and vibrational transition dipole moments (TDMs) can be deduced from analysis of centre line slope in 2DEV spectra [128]. In degenerate 2D optical spectroscopies, it is highly unusual to observe anti-correlated spectral features, as pump and probe pulses generally interrogate transition dipoles with the same, or very similar, vectors [146]. In 2DEV spectroscopy, correlations are made between electronic and vibrational TDMs, meaning the correlations in the 2DEV spectra should be sensitive to the relative orientation of the two TDMs. The positive or negative gradient of the CLSs in 2DEV spectra is dictated by whether the solvent environments are polarized along parallel or orthogonal vectors by the two different TDMs. With knowledge of the S_2_ ← S_0_ electronic TDM, which can be reliably predicted from time-dependent density functional calculations, the vectors associated with each normal mode, in tandem with the sign of the CLS in 2DEV spectra, allowed for the relative orientation of the two TDMs to be deduced, and make definitive assignments for some of excited state vibrational modes of bapo.

The C=C excited state feature at *ω*_3_ approximately 1590 cm^−1^ in the 2DEV spectra of β-apo-carotenal displayed in figure 6*b–d* is assigned to the C=C antisymmetric stretch on the photoexcited states. This feature blue-shifts and narrows along the *ω*_1_ axis as a function of increasing *t*_2_ time delay, and simultaneously the central infrared frequency also shifts to higher wavenumbers along *ω*_3_. The latter observation is associated with the changes in the force-constant associated with the vibrational mode, as molecules are transferred from the S_2_ surface to the S_1_ state, in line with *ab initio* calculations for shorter polyenes [147]. The bandwidth employed in this study (approx. 2000 cm^−1^ FWHM) is sufficient to excite one quanta of the high-frequency mid-IR probed after photoexcitation to the S_2_ state, and therefore the blue-shifting and narrowing along *ω*_1_ is interpreted as vibrational cooling of this mode in very anharmonic potential after ultrafast transfer to the S_1_ state. While no explicit modelling supports these specific observations, static 2DEV line shape calculations performed after the initial study support these hypotheses [131].

The most striking result from the 2DEV study of bapo was the long-lived correlation between electronic and vibrational line shape components for the C=C antisymmetric stretching mode, as probed via the *k*_e_ CLS, plotted in figure 6*b*–*d*. The decay of the *k*_e_ was fitted to an exponential decay with a 750 fs time constant, which is more than three times longer than the S_2_ lifetime. This is particularly surprising given the large reorganization on the S_1_ potential energy surface (*λ* approx. 8000 cm^−1^) that must occur after passage through the S_2_/S_1_ CI, which normally would be expected to quickly destroy any correlations. This means the solvent bath must remain frozen in the wake of change in electronic state and nuclear motions associated with this normal mode. Consequentially, the S_2_–S_1_ crossing must be ballistic and driven by a conical intersection close to the Franck–Condon region. The enhanced vibrational activity of this specific mode, and slow decay of the CLS relative to the S_2_ lifetime provide evidence to support the hypothesis that the antisymmetric C=C stretch of bapo is one of the tuning modes at the S_2_/S_1_ CI.

**Figure 7.**
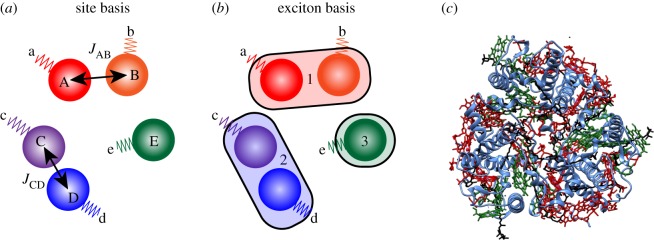
(*a*) Site basis of system containing five chromophores, labelled A through E, with each chromophore represented by an orb, (*b*) shaded boxes (1–3) depict the spatial delocalization for each exciton in the same system, (*c*) crystal structure of LHCII (PDB code 2BHW from Standfuss *et al.* [149]), where the protein is shown as a blue ribbon, and the skeletal structure of Chl-*a*, Chl-*b* and carotenoids are shown in red, green and black, respectively.

In dipole-coupled J-aggregates, such as light-harvesting antenna of plants, the excited electronic states are not localized to individual chromophores, but through dipole–dipole coupling delocalized across multiple pigments [148]. Figure 7*a*,*b* depicts the differences between the site (diabatic) and exciton (adiabatic) bases used to describe the energy structure of J-aggregates. The flow of energy between the different excitons has been revealed using 2DES for a large number of light-harvesting antenna [1,150–153]. However, establishing a link between exciton and site energies, and extraction of the intermolecular dipole coupling constants (*J*) has remained problematic without use of prior theoretical Hamiltonians or accompanying complex genetic mutant studies and polarization-dependent 2DES that help constrain the model [79,153].

Molecular vibrations are typically localized to specific physical chromophores, and therefore can act as a specific marker, or tag, for the exciton population on specific sites. This is illustrated in figure 7*a*,*b*, where individual chromophores are labelled with upper case letters and represented by orbs, vibrations localized to each chromophore are labelled in lower case letters, where *v*_a_ ≠ *v*_b_, etc. If the vibrational response could be correlated with broadband electronic excitation of all the associated exciton states, the time-dependent flow of energy between the various chromophores could in principle be followed.

A theoretical formalism for a coupled heterodimer system, using similar dipole coupling strengths, site reorganization energies and fluctuation correlation timescales to those previously derived for photosynthetic proteins, confirmed that 2DEV spectroscopy could be used to extract the uncoupled site energies and dipole coupling constants [129]. The key caveats are that vibrational frequencies associated with the two monomers must be distinct and so long as the excitonic splitting is resolvable in the inhomogeneous limit. The model requires that electronic coupling between the two moieties should not be strong enough to induce vibronic mixing in the vibrational Hamiltonian. This requirement is plausible for chlorophyll containing J-aggregates given the dipole coupling constants are modest, and the mid-IR active modes of chlorophyll (Chl) that can be investigated with 2DEV spectroscopy have very low associated Huang–Rhys factors [154,155]. Further the exciton energy splitting and vibrational modes probed must be very different. Again, for chlorophyll containing light-harvesting proteins this is reasonable as most of the exction energy gaps are an order of magnitude smaller than the mid-infrared active modes of Chl-*a/b* [156–160].

Simulated 2DEV spectra using Redfield theory in the secular approximation [161] showed that ground or excited state features could be used to extract the electronic site populations. The amplitude of each feature is proportional to the exciton population localized on each monomer, and thus the time-dependent site populations. This only requires the 2DEV spectral amplitudes and the ratios of the vibrational TDMs on the ground or electronically excited states. The former is the easiest to obtain from the linear absorption spectrum, whereas vibrational TDMs on electronically excited states require significant computational effort for multi-chromophore proteins such as light-harvesting complexes.

This theoretical work paved the way for 2DEV studies of LHCII [130]. LHCII is the most abundant pigment-protein complex on the planet, containing 14 chlorophyll molecules (eight Chl-*a*, six Chl-*b*) and four carotenoid moieties per monomer, and naturally forms a trimer (figure 7*c*). To date, no technique has been able to disentangle the site energies of pigments and the inter-pigment *J*-coupling constants, as prior 2DES measurements interrogate the adiabatic exciton structure [2,150,151]. As per the aforementioned theoretical study, the experimental approach is to use the high-frequency vibrational modes localized on Chl-*a* or Chl-*b* moieties as a proxy for spatial position inside the protein. The binding pockets for each chlorophyll molecule are inhomogeneous throughout the protein, which has two main effects: the electrostatic protein pockets shift the individual site energies of each chlorophyll moiety, which also modulates the inter-pigment *J*-coupling constants. Further, depending on the strength of the binding interactions and orientation of chlorophyll to proximal ligands, shifts in the mid-infrared vibrational frequencies can be induced.

The downside is that assignment of each LHCII vibrational feature is very difficult, as the protein-induced shifts are often very small, and LHCII contains 14 chlorophyll moieties per monomer. At present several features in the mid-infrared are readily assignable based on 2DEV studies of the individual Chl-*a* and Chl-*b* pigments in low-temperature glasses [162], and prior FTIR measurements [163]. Whereas assignments of many other features remain tentative and rely on previous LHCII Hamiltonians, which are based on fits to linear absorption, linear dichroism, circular dichroism, fluorescence spectra and transient absorption measurements [164].

The LHCII electronic linear absorption spectrum has previously been assigned to dominantly Chl-*a* (675 nm) and Chl-*b* (650 nm) excitons. For *t*_2_ > 50 ps, excited state energy transfer between the excitonic states is considered complete, and only a ‘thermal’ distribution of the lowest energy exciton states will be populated. The best theoretical Hamiltonians available predict that these excitons will be dominantly localized to Chl-*a* chromophores [164]. The 2DEV spectra reveal that this is not the case and that the lowest energy exciton states of LHCII have a significant population localized on Chl-*b* moieties via the presence of a peak that on the *ω*_1_ axes lies at 675 nm, but on the *ω*_3_ probe axes associated with Chl-*b* C=O stretching vibrations. The 2DEV study of LHCII has inspired further 2DES experiments and a re-evaluation and modifications to the LHCII Hamiltonian [165].

The most recent 2DEV spectroscopic studies [134] have used broadband octave-spanning mid-infrared probes (1600 cm^−1^ FWHM bandwidth) [115], which have the significant advantage of affording far more spectral bandwidth and far shorter pulse durations (21 fs) compared with OPA-generated mid-infrared pulses [53]. This means that the instrument response associated with 2DEV experiments is greatly reduced, enabling the technique to reach further back into the earliest time epoch of the excited state photochemical dynamics.

### 3.2. Two-dimensional vibrational–electronic spectroscopy

In tandem with the development of 2DEV spectroscopy, came the inception of a 2DVE spectroscopy, which interrogates the complementary extreme cross-peak between 2DIR and 2DES [121,166]. In this technique, mid-infrared laser pump (*k*_1_ and *k*_2_) prepares a population of a high-frequency vibration in either |*g*_0_〉 or |*g*_1_〉 states (figure 4*a*) that evolves in the *t*_2_ waiting time. The electronic probe pulse, *k*_3_, creates vibrational coherences between ground and excited electronic states, prior to emission and detection of the optical echo. 2DVE has offered insights into the nuclear-electronic dynamics between high-frequency ground state and electronic/vibronic Franck–Condon excitations. Courtney *et al*. used this spectroscopic technique to investigate vibronic coupling metal–metal charge transfer (MMCT) or ligand–metal charge transfer (LMCT) states of iron–ruthenium and iron-containing complexes [121,166]. The 2DVE spectra reveal signatures of features assigned to combination bands of C–N stretching modes coupled to low-frequency modes, which are strongly coupled to the MMCT and LMCT electronic transitions.

## 4. Two-dimensional fluorescence and photocurrent detected action spectroscopies

The 2D optical spectroscopies discussed thus far in this article employ three excitation pulses to induce the coherent nonlinear four-wave mixing (FWM) signals. Alternatively, instead of measuring the coherent signal, the third-order spectroscopic response of a system can be encoded in incoherent fields such as spontaneous fluorescence or photocurrents [98,167,168]. The same pulse sequence given in figure 2*a* is used; however, a fourth pulse, (*k*_4_) is used to convert the second coherence into a population state, which is amenable to detection via spontaneous fluorescence or photocurrent detection. The 2D optical correlation spectra can be encoded in the fluorescence or photocurrent by phase modulation of the four laser pulses, to retrieve the third-order nonlinear response of interest. Further, because the signal is detected purely in the time domain, both *t*_1_ and *t*_3_ delays must be scanned to construct the *ω*_1_–*ω*_3_ correlation maps. Despite the necessity to scan two time axes, 2D fluorescence spectroscopy (2DFS) can be implemented with rapid data acquisition using pulse shapers that change the pulse sequence at the repetition rate of the laser. The most recent studies report a root mean square error of less than 0.05 for 1 min of data acquisition and averaging for a single 2D spectrum [168].

There are several advantages of 2D action-based spectroscopies over conventional experiments that emit a coherent four-wave mixing signal: (i) Fluorescence is detected background free, and does not require heterodyne detection to retrieve the phase and amplitude information. Further, fluorescence detection is far more sensitive, as detectors capable of counting single photon emission events, such as photomultipliers or avalanche photodiodes, can be used with lock-in amplifiers. (ii) For molecules where photo-bleaching is an issue, such as DNA nucleobases, pump fluences required to generate the 2D signal can be greatly reduced by orders of magnitude. (iii) Fluorescence detection is insensitive to background noise generated from solvent scatter, which is especially a problem with ultraviolet pulses. (iv) There are no constraints on the geometry of the experiment and it can be performed in the fully collinear geometry. Such approaches could be used to report the 2D optical spectrum of sub-ensemble molecules, or even facilitate 2D spectra of single molecules. The recent fluorescence-encoded IR spectroscopy technique could also be applied to achieve these ambitions, where IR pump–probe measurements are encoded and read out by linear 2-photon fluorescence [169].

The Marcus group used 2DFS to determine the conformations of porphyrin dimers in phospholipid membranes [170,171], and a π-stacked dinucleotide [172]. Comparisons between 2DFS and 2DES spectra show the information content is very similar, but intensities for stimulated emission or excited state absorption features vary. Through a combination of simulations and experiments, it was shown that 2DFS and 2DES spectra are only identical if the final population state has a fluorescence quantum yield of 1. Self-quenching or fast non-radiative relaxation processes are the main causes for differences between the two sets of spectra, and can be exploited to deduce conformational structures [170,171].

While 2D action spectroscopies offer some practical advantages over coherently detected counterparts, they can also be used to extract the influence of photoexcitation on macroscopic properties, such as photocurrents, a key parameter used to determine the viability of photovoltaic cell efficiencies. The same pulse sequences used for 2DFS can also be used to detect photocurrent signals induced by four ultrafast laser pulses (2D photocurrent spectroscopy (2DPS)), and has recently been applied to semiconducting nanostructures, organic photovoltaic and PbS quantum dot containing photocells [173–175]. In the case of PbS quantum dots, 2DPS spectra contained very different spectral signatures to 2DFS data acquired under the same experimental conditions, for two different excitation wavelengths. 2DPS revealed signatures for multiple exciton generation via increasingly dispersive line shapes in the 2D correlation spectrum, whereas 2DFS spectra appeared insensitive to these processes [175].

## 5. Surface-specific two-dimensional infrared spectroscopy

As already discussed in this article, 2DIR spectroscopy is an incisive probe of vibrational coupling, macromolecular structure and protein folding of bulk structures. SFG is an even-order spectroscopic technique that is insensitive to centrosymmetric centres, such as bulk liquids, and sensitive to non-centrosymmetric centres such as surfaces interfaces. The Zanni group combined the surface-sensitivity of SFG spectroscopy with 2DIR spectroscopy to create a heterodyne detected 2D SFG technique, (HD) 2DSFG, and generate 2DIR correlation spectra of vibrations very near to, or at surface interfaces [176–178]. The pulse sequence is the same as shown in figure 3*a*, generated with a mid-infrared pulse shaper, but uses an additional narrowband visible pulse to up-convert the non-centrosymmetric infrared echo into the visible. The (HD) 2D SFG technique was used to investigate the conformation of a monolayer of AHP peptides on a gold surface [179]. 2D SFG was able, where linear SFG could not, to determine that the peptide adhered to the Au surface are α-helical and lie perpendicular to the plane of the gold surface, via the different anharmonic shifts extracted from 2D SFG and 2DIR spectra. In α-helical structures, normal modes are delocalized over multiple residues, and the associated amide-I vibrational potential is only slightly anharmonic. In randomly structured coils, the vibrational modes are far more localized and have larger associated anharmonic potentials. Thus, the greater anharmonic shifts observed in the 2DIR spectra, compared with (HD) 2D SFG measurements, demonstrated that AHP protein on the gold surface forms α-helical structures. Random coil residues are also detected via cross-peaks in the (HD) 2D SFG spectra via vibrational energy transfer between ordered and random coils, as the corresponding direct interrogation of these vibrations that would appear on the diagonal of the spectrum are forbidden.

## 6. Two-dimensional Raman and terahertz spectroscopies

Initial experiments by the Fleming and Miller groups to record two-dimensional Raman spectroscopy (2DRS) of liquids such as CS_2_ using non-resonant Raman pulses in six-wave mixing schemes were plagued by cascaded signals from sequential four-wave mixing processes [180–183]. Cascaded processes in these experiments originate from the third-order signal emission from one molecule, combining with incident laser beams, to induce a third-order emission from a second molecule. Most problematically, these signals emit in the same phase-matched direction as the desired fifth-order response 2D Raman signal.

Recently, the groups of Moran and Harel have implemented different excitation schemes to measure 2D Raman correlation spectra, with the majority of Raman interactions resonant with electronic states, and have demonstrated that such approaches ensure that the fifth-order 2D Raman response is dominant over sequential third-order cascaded processes [184–189].

Moran's group have used two different implementations of 2DRS, one where 2D correlation spectra are acquired exclusively in the time domain, and a second which involves a femtosecond-stimulated Raman scattering probe step which is amenable to mixed time-frequency detection [189]. The particular variant is chosen depending on the Raman frequencies of interest. 2DRS investigations have shown that wavepackets created in photoexcitation of ${\text{I}}_{3}^{-}$ in aqueous solution map into the ${\text{I}}_{2}^{-}$ photofragment product upon I–I bond cleavage [190]. Molesky *et al*. used 2DRS to reveal inhomogeneously broadened vibrations proximal to propionic acid side chains of water-ligated myoglobin, indicating that the side chains are conformationally flexible, with changes in structure driven by thermal fluctuations [186].

An alternative approach by the Harel group, takes advantage of the single shot 2DES method GRAPES, called gradient-assisted multidimensional electronic-Raman spectroscopy (GAMERS) [187,188]. A non-resonant Raman impulsive scattering pump that generates vibrational coherences on the ground electronic state, at time *t*_0_, is then proceeded via a typical 2DES pulse sequence. Data acquisition for a single *t*_2_ delay in GAMERS requires the *t*_0_ delay to be scanned, as the *t*_1_ delay is spatially encoded in the sample. The vast data content allows for frequency correlations to be made, via the appropriate Fourier-transform, between the *ω*_0_, *ω*_1_, *ω*_2_ and *ω*_3_ dimensions. Correlation maps between *ω*_0_ and *ω*_3_ for a dye molecule, IR 140 in solution reveal Raman active modes on ground or electronically excited states depending on which quadrature of the 2D electronic-Raman spectrum they appear in. Beating maps for specific Raman modes were constructed and overlaid with the *ω*_1_–*ω*_3_ 2DES correlation plots, revealing the parts of the electronic absorption spectrum associated with each Raman active vibration. The GAMERS spectroscopic technique is likely to prove decisive if applied to light-harvesting pigment-protein complexes, where arguments still persist whether observed oscillatory components of 2DES spectra arise from ground state or excited state vibrational wavepackets [132,191,192].

Mixed Raman-THz 2D spectroscopy experiments have been used to investigate the collective structural dynamics and intermolecular modes of water at ambient temperatures [193,194]. These studies highlight the appearance of an echo in the 2D Raman-THz spectra of liquid water that hints towards an inhomogeneous distribution of intermolecular hydrogen-bonding modes lives for up to 200 fs. This is significantly shorter than the lifetime of a single hydrogen bond (1 ps) and implies that extended water structures are not persistent at room temperature, bringing into question the conclusions drawn from prior X-ray scattering experiments. Recent 2D Raman-THz spectroscopic investigations of salt solutions revealed the correlation between bulk viscosities, where order in the water–hydrogen bonding network is far longer lived than in pure water, and that the cations can have a significant influence on the water network structure and associated reorganization timescales [195]. 2D THz-Raman experiments were also used to elucidate the off-diagonal anharmonic coupling between low-frequency Raman modes of halogenated liquids [196].

The wider availability of THz pulses, and free-space electro-optic sampling which fully characterizes the absolute phase and amplitude of THz pulses has allowed nonlinear ultrafast 2D THz spectroscopies to explore a variety of material properties. 2D THz spectroscopy has been used to investigate intersubband transitions in double quantum well systems, where a 2π Rabi flop is observed from coupling of the large dipole moment of the intersubband transition to the THz radiation [117,197]. For graphene, it was demonstrated that even at small THz driving fields, the nonlinear response is outside of the non-perturbative limit, and that coupling of carriers to the electromagnetic field dominated over any other scattering process [198]. Very recently, 2D THz spectroscopy has been used to reveal the nonlinear response of magnons, collective spin waves, in anti-ferromagnetic crystals. This work paves the way to investigate spin interactions between anti-ferromagnetic and ferromagnetic order switching [199]. Investigations of pure liquids or molecular-based systems with 2D THz spectroscopy have yet to be realized, because of the far lower nonlinearities associated with these molecular systems compared with solid-state materials, and insufficient THz pulse energies [117]. Hopefully, future developments of higher power plasma THz sources will provide sufficient pulse energies for 2D THz investigations of low-frequency intermolecular phonon modes of pure liquids and solute molecules.

## 7. Future research directions

Multidimensional optical spectroscopies now represent some of the premier tools for studying condensed phase dynamics of chemical, biological and nanomaterial systems. Since their first inception [40,44], they have rapidly evolved from original infrared and optical domains, spawning three-dimensional counterparts, [37,200] and can interrogate ultrafast dynamics throughout the entire electromagnetic spectrum. Theoretical calculations by Mukamel predict that future 2D spectroscopies using X-ray pulses could directly probe non-Born–Oppenheimer dynamics involving CIs [201–203].

### 7.1. Two-dimensional electronic–vibrational spectroscopy with ultraviolet excitation

Coupling broadband ultraviolet light with vibrational probes is one logical extension of 2DEV for the study of non-adiabatic dynamics in ultraviolet chromophores such as nucleobases, cytochromes, photoswitches and smaller polyenes that are more amenable to *ab initio* studies. In the majority of these systems, CIs near to the vertical Franck–Condon region are far more commonplace than in visible chromophores and almost always inevitably lead to strongly coupled electronic–nuclear motion that 2DEV spectroscopy is well placed to interrogate [128,139,204,205]. The necessary technological components already exist: ultrashort broadband UV sources are readily available [184,206], 2DES has been performed with ultraviolet laser pulses and thus the significant technological challenge of creating phase-locked UV pulse pairs has been surmounted [95,207–211].

Photoexcitation of UV chromophores is normally associated with large nuclear displacements, which in particular cases may drive wavepacket dynamics associated with the high-frequency vibrational modes. If the same frequencies can be probed with mid-infrared pulses, this will inevitably lead to an increased number of 2DEV spectroscopic pathways than depicted in figure 4, as probed via 1 → 0 or 2 ← 1 vibrational transitions on the excited state. Depending on the coherences generated, some of these pathways may very well oscillate in the waiting time.

### 7.2. Spatially resolved two-dimensional electronic spectroscopy

The work of Baiz and Tokmakoff demonstrated that a fully collinear 2DIR pulse train could be successfully combined with confocal microscopy, to realize 2DIR with micrometre spatial resolution [212]. Such experiments were quickly followed by wide-field 2DIR imaging by the Zanni group [213,214]. Such spectroscopies, even with spatial resolution above the diffraction limit, provide opportunities to explore the infrared signatures of heterogeneous biological samples such as tissues *in vivo*. Furthermore, the reduced spot size means samples experience far higher peak powers, which generates larger nonlinear signals which may afford reduced acquisition times. While many forays have been made to perform visible pump–probe microscopy experiments [215–218], which with near-field delivery of pulses to samples can attain spatial resolutions below the diffraction limit [219], to date no experiment has reported spatially resolved 2DES measurements. If applied to photovoltaic materials, one imagines that spatially resolved 2DES could reveal additional key information about the influence of spatial morphology on energy transfer or charge-separation dynamics. Visible pulses are potentially more damaging to samples than mid-infrared, and can induce photo-bleaching, as already demonstrated for confocal transient absorption measurements. This will be more problematic for confocal 2DES because of the increased acquisition times due to the requirement that *t*_1_ must be scanned (cf. just *t*_2_ in pump–probe). But with the proven success of compressed sensing to 2D correlation spectroscopies and rapid phase cycling, it is foreseeable that these problems can be surmounted in the near future. An alternative approach would be to employ 2D action spectroscopies such as 2DFS, where far lower pump intensities can be used [168].

### 7.3. Fully chiral two-dimensional electronic spectroscopy

The interpretation of oscillatory signals observed in 2DES data for photosynthetic light-harvesting proteins have attracted a lot of controversy [1,3,4,72,132,191,220,221]. The ongoing debate revolves around whether these signals originate from purely electronic, vibrational or mixed vibronic effects. To date, experiments have yet to be able to definitively differentiate between signals arising from these processes. Recently, theoretical calculations from the Collini and Olaya-Castro groups have shown that fully chiral 2DES experiments could be used to differentiate between purely electronic, vibronic and vibrational coherences [222,223]. To date, only experiments using either pump or probe pulses that are elliptically polarized have been demonstrated for 2DES [224,225]. If experimentally realized, fully chiral 2DES could answer this almost 10-year-old debate. Such a technique would also be incredibly sensitive, as all chiral-specific spectroscopies are, to changes in conformational structure involving chiral centres induced by excited state dynamics.

## Data accessibility

Isotropic 2DIR data for MAPbI_3_ are available for download from the University of Bristol's Research Data Storage Facility: https://doi.org/10.5523/bris.1qlc372dvn9in2gtnb7vpxfait.

## References

[1] Engel GS, Calhoun TR, Read EL, Ahn T-K, Mancal T, Cheng Y-C, Blankenship RE, Fleming GR 2007 Evidence for wavelike energy transfer through quantum coherence in photosynthetic systems. Nature 446, 782–786. (doi:10.1038/nature05678) 17429397 10.1038/nature05678

[2] Calhoun TR, Ginsberg NS, Schlau-Cohen GS, Cheng Y-C, Ballottari M, Bassi R, Fleming GR 2009 Quantum coherence enabled determination of the energy landscape in light-harvesting complex II. J. Phys. Chem. B 113, 16 291–16 295. (doi:10.1021/jp908300c) 10.1021/jp908300c 20014871

[3] Collini E, Wong CY, Wilk KE, Curmi PMG, Brumer P, Scholes GD 2010 Coherently wired light-harvesting in photosynthetic marine algae at ambient temperature. Nature 463, 644–647. (doi:10.1038/nature08811) 20130647 10.1038/nature08811

[4] Panitchayangkoon G, Hayes D, Fransted KA, Caram JR, Harel E, Wen J, Blankenship RE, Engel GS 2010 Long-lived quantum coherence in photosynthetic complexes at physiological temperature. Proc. Natl Acad. Sci. USA 107, 12 766–12 770. (doi:10.1073/pnas.1005484107) 10.1073/pnas.1005484107 PMC2919932 20615985

[5] Fuller FD et al. 2014 Vibronic coherence in oxygenic photosynthesis. Nat. Chem. 6, 706–711. (doi:10.1038/nchem.2005) 25054941 10.1038/nchem.2005

[6] Romero E, Novoderezhkin VI, van Grondelle R 2017 Quantum design of photosynthesis for bio-inspired solar-energy conversion. Nature 543, 355–365. (doi:10.1038/nature22012) 28300093 10.1038/nature22012

[7] Scholes GD et al. 2017 Using coherence to enhance function in chemical and biophysical systems. Nature 543, 647–656. (doi:10.1038/nature21425) 28358065 10.1038/nature21425

[8] Song Y, Clafton SN, Pensack RD, Kee TW, Scholes GD 2014 Vibrational coherence probes the mechanism of ultrafast electron transfer in polymer–fullerene blends. Nat. Commun. 5, 4933 (doi:10.1038/ncomms5933) 25215959 10.1038/ncomms5933

[9] Falke SM et al. 2014 Coherent ultrafast charge transfer in an organic photovoltaic blend. Science 344, 1001–1005. (doi:10.1126/science.1249771) 24876491 10.1126/science.1249771

[10] De Sio A et al. 2016 Tracking the coherent generation of polaron pairs in conjugated polymers. Nat. Commun. 7, 13 742–13 748. (doi:10.1038/ncomms13742) 10.1038/ncomms13742 PMC5155154 27929115

[11] Brédas J-L, Sargent EH, Scholes GD 2016 Photovoltaic concepts inspired by coherence effects in photosynthetic systems. Nat. Mater. 16, 35–44. (doi:10.1038/nmat4767) 27994245 10.1038/nmat4767

[12] Cundiff ST 2008 Coherent spectroscopy of semiconductors. Opt. Express 16, 4639 (doi:10.1364/OE.16.004639) 18542562 10.1364/oe.16.004639

[13] Stone KW, Gundogdu K, Turner DB, Li X, Cundiff ST, Nelson KA 2009 Two-quantum 2D FT electronic spectroscopy of biexcitons in GaAs quantum wells. Science 324, 1169–1173. (doi:10.1126/science.1170274) 19478176 10.1126/science.1170274

[14] Cundiff ST, Bristow AD, Siemens M, Li H, Moody G, Karaiskaj D, Dai X, Zhang T 2012 Optical 2-D Fourier transform spectroscopy of excitons in semiconductor nanostructures. IEEE J. Select. Topics Quantum Electron. 18, 318–328. (doi:10.1109/JSTQE.2011.2123876)

[15] Huxter VM, Oliver TAA, Budker D, Fleming GR 2013 Vibrational and electronic dynamics of nitrogen–vacancy centres in diamond revealed by two-dimensional ultrafast spectroscopy. Nat. Phys. 9, 744–749. (doi:10.1038/nphys2753)

[16] Bakulin AA et al. 2015 Real-time observation of organic cation reorientation in methylammonium lead iodide perovskites. J. Phys. Chem. Lett. 6, 3663–3669. (doi:10.1021/acs.jpclett.5b01555) 26722739 10.1021/acs.jpclett.5b01555

[17] Chung HS, Ganim Z, Jones KC, Tokmakoff A 2007 Transient 2D IR spectroscopy of ubiquitin unfolding dynamics. Proc. Natl Acad. Sci. USA 104, 14 237–14 242. (doi:10.1073/pnas.0700959104) 10.1073/pnas.0700959104 PMC1964855 17551015

[18] Strasfeld DB, Ling YL, Shim S-H, Zanni MT 2008 Tracking fiber formation in human islet amyloid polypeptide with automated 2D-IR spectroscopy. J. Am. Chem. Soc. 130, 6698–6699. (doi:10.1021/ja801483n) 18459774 10.1021/ja801483n PMC3209517

[19] Shim S-H, Gupta R, Ling YL, Strasfeld DB, Raleigh DP, Zanni MT 2009 Two-dimensional IR spectroscopy and isotope labeling defines the pathway of amyloid formation with residue-specific resolution. Proc. Natl Acad. Sci. USA 106, 6614–6619. (doi:10.1073/pnas.0805957106) 19346479 10.1073/pnas.0805957106 PMC2672516

[20] Chung JK, Thielges MC, Fayer MD 2011 Dynamics of the folded and unfolded villin headpiece (HP35) measured with ultrafast 2D IR vibrational echo spectroscopy. Proc. Natl Acad. Sci. USA 108, 3578–3583. (doi:10.1073/pnas.1100587108) 21321226 10.1073/pnas.1100587108 PMC3048147

[21] King JT, Kubarych KJ 2012 Site-specific coupling of hydration water and protein flexibility studied in solution with ultrafast 2D-IR spectroscopy. J. Am. Chem. Soc. 134, 18 705–18 712. (doi:10.1021/ja307401r) 10.1021/ja307401r 23101613

[22] Ghosh A, Qiu J, DeGrado WF, Hochstrasser RM 2011 Tidal surge in the M2 proton channel, sensed by 2D IR spectroscopy. Proc. Natl Acad. Sci. USA 108, 6115–6120. (doi:10.1073/pnas.1103027108) 21444789 10.1073/pnas.1103027108 PMC3076828

[23] Pagano P, Guo Q, Kohen A, Cheatum CM 2016 Oscillatory enzyme dynamics revealed by two-dimensional infrared spectroscopy. J. Phys. Chem. Lett. 7, 2507–2511. (doi:10.1021/acs.jpclett.6b01154) 27305279 10.1021/acs.jpclett.6b01154 PMC4939886

[24] Brixner T, Mancal T, Stiopkin IV, Fleming GR 2004 Phase-stabilized two-dimensional electronic spectroscopy. J. Chem. Phys. 121, 4221–4236. (doi:10.1063/1.1776112) 15332970 10.1063/1.1776112

[25] Christensson N, Milota F, Hauer J, Sperling J, Bixner O, Nemeth A, Kauffmann HF 2011 High frequency vibrational modulations in two-dimensional electronic spectra and their resemblance to electronic coherence signatures. J. Phys. Chem. B 115, 5383–5391. (doi:10.1021/jp109442b) 21329370 10.1021/jp109442b

[26] Lukes V, Christensson N, Milota F, Kauffmann HF 2011 Electronic ground state conformers of β-carotene and their role in ultrafast spectroscopy. Chem. Phys. Lett. 506, 122–127. (doi:10.1016/j.cplett.2011.02.060)

[27] Calhoun TR, Davis JA, Graham MW 2012 The separation of overlapping transitions in β-carotene with broadband 2D electronic spectroscopy. Chem. Phys. Lett. 523, 1–5. (doi:10.1016/j.cplett.2011.10.051)

[28] Woutersen S, Mu Y, Stock G, Hamm P 2001 Hydrogen-bond lifetime measured by time-resolved 2D-IR spectroscopy: N-methylacetamide in methanol. Chem. Phys. 266, 137–147. (doi:10.1016/s0301-0104(01)00224-5)

[29] Kwac K, Cho M 2003 Two-color pump− probe spectroscopies of two-and three-level systems: 2-dimensional line shapes and solvation dynamics. J. Phys. Chem. A 107, 5903–5912. (doi:10.1021/jp034727w)

[30] Zheng J 2005 Ultrafast dynamics of solute-solvent complexation observed at thermal equilibrium in real time. Science 309, 1338–1343. (doi:10.1126/science.1116213) 16081697 10.1126/science.1116213

[31] Kwak K, Park S, Finkelstein IJ, Fayer MD 2007 Frequency-frequency correlation functions and apodization in two-dimensional infrared vibrational echo spectroscopy: a new approach. J. Chem. Phys. 127, 124503 (doi:10.1063/1.2772269) 17902917 10.1063/1.2772269

[32] Kwak K, Rosenfeld DE, Fayer MD 2008 Taking apart the two-dimensional infrared vibrational echo spectra: more information and elimination of distortions. J. Chem. Phys. 128, 204505 (doi:10.1063/1.2927906) 18513030 10.1063/1.2927906

[33] Ramasesha K, De Marco L, Mandal A, Tokmakoff A 2013 Water vibrations have strongly mixed intra- and intermolecular character. Nat. Chem. 5, 935–940. (doi:10.1038/nchem.1757) 24153371 10.1038/nchem.1757

[34] Ruetzel S, Diekmann M, Nuernberger P, Walter C, Engels B, Brixner T 2014 Multidimensional spectroscopy of photoreactivity. Proc. Natl Acad. Sci. USA 111, 4764–4769. (doi:10.1073/pnas.1323792111) 24639540 10.1073/pnas.1323792111 PMC3977289

[35] Ren Z, Ivanova AS, Couchot-Vore D, Garrett-Roe S 2014 Ultrafast structure and dynamics in ionic liquids: 2D-IR spectroscopy probes the molecular origin of viscosity. J. Phys. Chem. Lett. 5, 1541–1546. (doi:10.1021/jz500372f) 26270093 10.1021/jz500372f

[36] Brinzer T, Berquist EJ, Ren Z, Dutta S, Johnson CA, Krisher CS, Lambrecht DS, Garrett-Roe S 2015 Ultrafast vibrational spectroscopy (2D-IR) of CO_2_ in ionic liquids: carbon capture from carbon dioxide's point of view. J. Chem. Phys. 142, 212425 (doi:10.1063/1.4917467) 26049445 10.1063/1.4917467

[37] Garrett-Roe S, Perakis F, Rao F, Hamm P 2011 Three-dimensional infrared spectroscopy of isotope-substituted liquid water reveals heterogeneous dynamics. J. Phys. Chem. B 115, 6976–6984. (doi:10.1021/jp201989s) 21561076 10.1021/jp201989s

[38] Dahms F, Fingerhut BP, Nibbering ETJ, Pines E, Elsaesser T 2017 Large-amplitude transfer motion of hydrated excess protons mapped by ultrafast 2D IR spectroscopy. Science 357, 491–495. (doi:10.1126/science.aan5144) 28705988 10.1126/science.aan5144

[39] Roberts ST, Loparo JJ, Tokmakoff A 2006 Characterization of spectral diffusion from two-dimensional line shapes. J. Chem. Phys. 125, 084502 (doi:10.1063/1.2232271) 16965024 10.1063/1.2232271

[40] Hamm P, Lim M, Hochstrasser RM 1998 Structure of the amide I band of peptides measured by femtosecond nonlinear-infrared spectroscopy. J. Phys. Chem. B 102, 6123–6138. (doi:10.1021/jp9813286)

[41] Hamm P, Lim M, DeGrado WF, Hochstrasser RM 1999 The two-dimensional IR nonlinear spectroscopy of a cyclic penta-peptide in relation to its three-dimensional structure. Proc. Natl Acad. Sci. USA 96, 2036–2041. (doi:10.1073/pnas.96.5.2036) 10051590 10.1073/pnas.96.5.2036 PMC26732

[42] Golonzka O, Khalil M, Demirdöven N, Tokmakoff A 2001 Vibrational anharmonicities revealed by coherent two-dimensional infrared spectroscopy. Phys. Rev. Lett. 86, 2154–2157. (doi:10.1103/PhysRevLett.86.2154) 11289878 10.1103/PhysRevLett.86.2154

[43] Asbury JB, Steinel T, Stromberg C, Gaffney KJ, Piletic IR, Fayer MD 2003 Hydrogen bond breaking probed with multidimensional stimulated vibrational echo correlation spectroscopy. J. Chem. Phys. 119, 12 981–12 997. (doi:10.1063/1.1627762)

[44] Hybl JD, Albrecht Ferro A, Jonas DM 2001 Two-dimensional Fourier transform electronic spectroscopy. J. Chem. Phys. 115, 6606–6622. (doi:10.1063/1.1398579) 10.1063/1.4867996 24697446

[45] Cowan ML, Ogilvie JP, Miller RJD 2004 Two-dimensional spectroscopy using diffractive optics based phased-locked photon echoes. Chem. Phys. Lett. 386, 184–189. (doi:10.1016/j.cplett.2004.01.027)

[46] Nibbering E, Wiersma D, Duppen K 1991 Femtosecond non-Markovian optical dynamics in solution. Phys. Rev. Lett. 66, 2464–2467. (doi:10.1103/PhysRevLett.66.2464) 10043495 10.1103/PhysRevLett.66.2464

[47] Cho M, Scherer NF, Fleming GR, Mukamel S 1992 Photon echoes and related four-wave-mixing spectroscopies using phase-locked pulses. J. Chem. Phys. 96, 5618 (doi:10.1063/1.462686)

[48] Tokmakoff A, Zimdars D, Sauter B, Francis RS, Kwok AS, Fayer MD 1994 Vibrational photon echoes in a liquid and glass: room temperature to 10 K. J. Chem. Phys. 101, 1741–1744. (doi:10.1063/1.467731)

[49] Cho M, Fleming GR 1993 Photon echo measurements in liquids: numerical calculations with model systems. J. Chem. Phys. 98, 2848–2859. (doi:10.1063/1.464114)

[50] de Boeij WP, Pshenichnikov MS, Wiersma DA 1998 Heterodyne-detected stimulated photon echo: applications to optical dynamics in solution. Chem. Phys. 233, 287–309. (doi:10.1016/s0301-0104(98)00084-6)

[51] Ohta K, Larsen DS, Yang M, Fleming GR 2001 Influence of intramolecular vibrations in third-order, time-domain resonant spectroscopies. II. Numerical calculations. J. Chem. Phys. 114, 8020 (doi:10.1063/1.1359241)

[52] Wilhelm T, Piel J, Riedle E 1997 Sub-20-fs pulses tunable across the visible from a blue-pumped single-pass noncollinear parametric converter. Opt. Lett. 22, 1494–1496. (doi:10.1364/OL.22.001494) 18188279 10.1364/ol.22.001494

[53] Hamm P, Kaindl RA, Stenger J 2000 Noise suppression in femtosecond mid-infrared light sources. Opt. Lett. 25, 1798 (doi:10.1364/OL.25.001798) 18066348 10.1364/ol.25.001798

[54] Cerullo G, De Silvestri S 2003 Ultrafast optical parametric amplifiers. Rev. Sci. Instrum. 74, 1–18. (doi:10.1063/1.1523642)

[55] Kobayashi T, Shirakawa A 2000 Tunable visible and near-infrared pulse generator in a 5 fs regime. Appl. Phys. B 70, S239–S246. (doi:10.1007/s003400000325)

[56] Mukamel S 2000 Multidimensional femtosecond correlation spectroscopies of electronic and vibrational excitations. Annu. Rev. Phys. Chem. 51, 691–729. (doi:10.1146/annurev.physchem.51.1.691) 11031297 10.1146/annurev.physchem.51.1.691

[57] Jonas DM 2003 Two-dimensional femtosecond spectroscopy. Annu. Rev. Phys. Chem. 54, 425–463. (doi:10.1146/annurev.physchem.54.011002.103907) 12626736 10.1146/annurev.physchem.54.011002.103907

[58] Hochstrasser RM 2007 Multidimensional ultrafast spectroscopy special feature: two-dimensional spectroscopy at infrared and optical frequencies. Proc. Natl Acad. Sci. USA 104, 14 190–14 196. (doi:10.1073/pnas.0704079104) 10.1073/pnas.0706002104 PMC1964836 17724335

[59] Ogilvie JP, Kubarych KJ 2009 Multidimensional electronic and vibrational spectroscopy: an ultrafast probe of molecular relaxation and reaction dynamics, 1st edn Amsterdam, The Netherlands: Elsevier Inc.

[60] Milota F, Sperling J, Nemeth A, Mancal T, Kauffmann HF 2009 Two-dimensional electronic spectroscopy of molecular excitons. Acc. Chem. Res. 42, 1364–1374. (doi:10.1021/ar800282e) 19673525 10.1021/ar800282e

[61] Stone KW, Turner DB, Gundogdu K, Cundiff ST, Nelson KA 2009 Exciton–exciton correlations revealed by two-quantum, two-dimensional Fourier transform optical spectroscopy. Acc. Chem. Res. 42, 1452–1461. (doi:10.1021/ar900122k) 19691277 10.1021/ar900122k

[62] Fayer MD 2009 Dynamics of liquids, molecules, and proteins measured with ultrafast 2D IR vibrational echo chemical exchange spectroscopy. Annu. Rev. Phys. Chem. 60, 21–38. (doi:10.1146/annurev-physchem-073108-112712) 18851709 10.1146/annurev-physchem-073108-112712

[63] Ginsberg NS, Cheng Y-C, Fleming GR 2009 Two-dimensional electronic spectroscopy of molecular aggregates. Acc. Chem. Res. 42, 1352–1363. (doi:10.1021/ar9001075) 19691358 10.1021/ar9001075

[64] Thielges MC, Fayer MD 2012 Protein dynamics studied with ultrafast two-dimensional infrared vibrational echo spectroscopy. Acc. Chem. Res. 45, 1866–1874. (doi:10.1021/ar200275k) 22433178 10.1021/ar200275k PMC3389584

[65] Dawlaty JM, Ishizaki A, De AK, Fleming GR 2012 Microscopic quantum coherence in a photosynthetic-light-harvesting antenna. Phil. Trans. R. Soc. A 370, 3672–3691. (doi:10.1063/1.2978381) 22753820 10.1098/rsta.2011.0207

[66] Cho M 2013 Coherent two-dimensional optical spectroscopy. Chem. Rev. 108, 1331–1418. (doi:10.1021/cr078377b) 10.1021/cr078377b 18363410

[67] Nuernberger P, Ruetzel S, Brixner T 2015 Multidimensional electronic spectroscopy of photochemical reactions. Angew. Chem. Int. Ed. 54, 11 368–11 386. (doi:10.1002/anie.201502974) 10.1002/anie.201502974 26382095

[68] Fuller FD, Ogilvie JP 2015 Experimental implementations of two-dimensional Fourier transform electronic spectroscopy. Annu. Rev. Phys. Chem. 66, 667–690. (doi:10.1146/annurev-physchem-040513-103623) 25664841 10.1146/annurev-physchem-040513-103623

[69] Ghosh A, Ostrander JS, Zanni MT 2017 Watching proteins wiggle: mapping structures with two-dimensional infrared spectroscopy. Chem. Rev. 117, 10 726–10 759. (doi:10.1021/acs.chemrev.6b00582) 10.1021/acs.chemrev.6b00582 PMC5500453 28060489

[70] Nee MJ, Baiz CR, Anna JM, McCanne R, Kubarych KJ 2008 Multilevel vibrational coherence transfer and wavepacket dynamics probed with multidimensional IR spectroscopy. J. Chem. Phys. 129, 084503 (doi:10.1063/1.2969900) 19044831 10.1063/1.2969900

[71] Collini E, Scholes GD 2009 Coherent intrachain energy migration in a conjugated polymer at room temperature. Science 323, 369–373. (doi:10.1126/science.1164016) 19150843 10.1126/science.1164016

[72] Hayes D, Griffin GB, Engel GS 2013 Engineering coherence among excited states in synthetic heterodimer systems. Science 340, 1431–1434. (doi:10.1126/science.1233828) 23599263 10.1126/science.1233828

[73] Marroux HJB, Orr-Ewing AJ 2016 Distinguishing population and coherence transfer pathways in a metal dicarbonyl complex using pulse-shaped two-dimensional infrared spectroscopy. J. Phys. Chem. B 120, 4125–4130. (doi:10.1021/acs.jpcb.6b02979) 27070852 10.1021/acs.jpcb.6b02979

[74] Shim S-H, Strasfeld DB, Ling YL, Zanni MT 2007 Automated 2D IR spectroscopy using a mid-IR pulse shaper and application of this technology to the human islet amyloid polypeptide. Proc. Natl Acad. Sci. USA 104, 14 197–14 202. (doi:10.1073/pnas.0700804104) 10.1073/pnas.0700804104 PMC1964818 17502604

[75] Fuller FD, Wilcox DE, Ogilvie JP 2014 Pulse shaping based two-dimensional electronic spectroscopy in a background free geometry. Opt. Express 22, 1018 (doi:10.1364/OE.22.001018) 24515061 10.1364/OE.22.001018

[76] Hochstrasser RM 2001 Two-dimensional IR-spectroscopy: polarization anisotropy effects. Chem. Phys. 266, 273–284. (doi:10.1016/S0301-0104(01)00232-4)

[77] Zanni MT, Ge NH, Kim YS, Hochstrasser RM 2001 Two-dimensional IR spectroscopy can be designed to eliminate the diagonal peaks and expose only the crosspeaks needed for structure determination. Proc. Natl Acad. Sci. USA 98, 11 265–11 270. (doi:10.1073/pnas.201412998) 11562493 10.1073/pnas.201412998 PMC58718

[78] Read EL, Engel GS, Calhoun TR, Mancal T, Ahn T-K, Blankenship RE, Fleming GR 2007 Cross-peak-specific two-dimensional electronic spectroscopy. Proc. Natl Acad. Sci. USA 104, 14 203–14 208. (doi:10.1073/pnas.0701201104) 10.1073/pnas.0701201104 PMC1964816 17548830

[79] Schlau-Cohen GS, Calhoun TR, Ginsberg NS, Ballottari M, Bassi R, Fleming GR 2010 Spectroscopic elucidation of uncoupled transition energies in the major photosynthetic light-harvesting complex, LHCII. Proc. Natl Acad. Sci. USA 107, 13 276–13 281. (doi:10.1073/pnas.1006230107) 10.1073/pnas.1006230107 PMC2922122 20622154

[80] Brixner T, Stiopkin IV, Fleming GR 2004 Tunable two-dimensional femtosecond spectroscopy. Opt. Lett. 29, 884–886. (doi:10.1364/OL.29.000884) 15119410 10.1364/ol.29.000884

[81] Zhang T, Borca C, Li X, Cundiff S 2005 Optical two-dimensional Fourier transform spectroscopy with active interferometric stabilization. Opt. Express 13, 7432–7441. (doi:10.1364/OPEX.13.007432) 19498768 10.1364/opex.13.007432

[82] Zhu W et al. 2017 Broadband two-dimensional electronic spectroscopy in an actively phase stabilized pump-probe configuration. Opt. Express 25, 21115 (doi:10.1364/OE.25.021115) 29041519 10.1364/OE.25.021115

[83] Bristow AD, Karaiskaj D, Dai X, Zhang T, Carlsson C, Hagen KR, Jimenez R, Cundiff ST 2009 A versatile ultrastable platform for optical multidimensional Fourier-transform spectroscopy. Rev. Sci. Instrum. 80, 073108 (doi:10.1063/1.3184103) 19655944 10.1063/1.3184103

[84] Milota F, Lincoln CN, Hauer J 2013 Precise phasing of 2D-electronic spectra in a fully non-collinear phase-matching geometry. Opt. Express 21, 15 904–15 911. (doi:10.1364/OE.21.015904) 10.1364/OE.21.015904 23842377

[85] Anna JM, Ostroumov EE, Maghlaoui K, Barber J, Scholes GD 2012 Two-dimensional electronic spectroscopy reveals ultrafast downhill energy transfer in photosystem I trimers of the cyanobacterium thermosynechococcus elongatus. J. Phys. Chem. Lett. 3, 3677–3684. (doi:10.1021/jz3018013) 26291095 10.1021/jz3018013

[86] DeCamp MF, Deflores LP, Jones KC, Tokmakoff A 2007 Single-shot two-dimensional infrared spectroscopy. Opt. Express 15, 233–241. (doi:10.1364/OE.15.000233) 19532239 10.1364/oe.15.000233

[87] Harel E, Fidler AF, Engel GS 2010 Real-time mapping of electronic structure with single-shot two-dimensional electronic spectroscopy. Proc. Natl Acad. Sci. USA 107, 16 444–16 447. (doi:10.1073/pnas.1007579107) 20810917 10.1073/pnas.1007579107 PMC2944741

[88] Harel E, Fidler AF, Engel GS 2011 Single-shot gradient-assisted photon echo electronic spectroscopy. J. Phys. Chem. A 115, 3787–3796. (doi:10.1021/jp107022f) 21090733 10.1021/jp107022f

[89] Donaldson PM, Strzalka H, Hamm P 2012 High sensitivity transient infrared spectroscopy: a UV/visible transient grating spectrometer with a heterodyne detected infrared probe. Opt. Express 20, 12 761–12 770. (doi:10.1364/OE.20.012761) 10.1364/OE.20.012761 22714305

[90] Gallagher Faeder SM, Jonas DM 1999 Two-dimensional electronic correlation and relaxation spectra: theory and model calculations. J. Phys. Chem. A 103, 10 489–10 505. (doi:10.1021/jp9925738)

[91] Myers JA, Lewis KLM, Tekavec PF, Ogilvie JP 2008 Two-color two-dimensional Fourier transform electronic spectroscopy with a pulse-shaper. Opt. Express 16, 17 420–17 428. (doi:10.1364/OE.16.017420) 10.1364/oe.16.017420 18958024

[92] Cheng Y-C, Fleming GR 2008 Coherence quantum beats in two-dimensional electronic spectroscopy. J. Phys. Chem. A 112, 4254–4260. (doi:10.1021/jp7107889) 18376878 10.1021/jp7107889

[93] Cheng Y-C, Fleming GR 2009 Dynamics of light harvesting in photosynthesis. Annu. Rev. Phys. Chem. 60, 241–262. (doi:10.1146/annurev.physchem.040808.090259) 18999996 10.1146/annurev.physchem.040808.090259

[94] Réhault J, Maiuri M, Oriana A, Cerullo G 2014 Two-dimensional electronic spectroscopy with birefringent wedges. Rev. Sci. Instrum. 85, 123107 (doi:10.1063/1.4902938) 25554272 10.1063/1.4902938

[95] Borrego-Varillas R, Oriana A, Ganzer L, Trifonov A, Manzoni C 2016 Two-dimensional electronic spectroscopy in the ultraviolet by a birefringent delay line. Opt. Express 24, 28 491–28 499. (doi:10.1364/OE.24.028491) 10.1364/OE.24.028491 27958492

[96] Shim S-H, Zanni MT 2009 How to turn your pump–probe instrument into a multidimensional spectrometer: 2D IR and vis spectroscopies via pulse shaping. Phys. Chem. Chem. Phys. 11, 748 (doi:10.1039/b813817f) 19290321 10.1039/b813817f PMC2821705

[97] Turner DB, Stone KW, Gundogdu K, Nelson KA 2011 Invited article: the coherent optical laser beam recombination technique (COLBERT) spectrometer: coherent multidimensional spectroscopy made easier. Rev. Sci. Instrum. 82, 081301 (doi:10.1063/1.3624752) 21895226 10.1063/1.3624752

[98] Tian P, Keusters D, Suzaki Y, Warren WS 2003 Femtosecond phase-coherent two-dimensional spectroscopy. Science 300, 1553–1555. (doi:10.1126/science.1083433) 12791987 10.1126/science.1083433

[99] Shim SH, Strasfeld DB, Zanni MT 2006 Generation and characterization of phase and amplitude shaped femtosecond mid-IR pulses. Opt. Express 14, 13 120–13 130. (doi:10.1364/OE.14.013120) 10.1364/oe.14.013120 19532209

[100] Zhang Z, Wells KL, Hyland EWJ, Tan H-S 2012 Phase-cycling schemes for pump–probe beam geometry two-dimensional electronic spectroscopy. Chem. Phys. Lett. 550, 156–161. (doi:10.1016/j.cplett.2012.08.037)

[101] Sanders JN, Saikin SK, Mostame S, Andrade X, Widom JR, Marcus AH, Aspuru-Guzik A 2012 Compressed sensing for multidimensional spectroscopy experiments. J. Phys. Chem. Lett. 3, 2697–2702. (doi:10.1021/jz300988p) 26295894 10.1021/jz300988p

[102] Humston JJ, Bhattacharya I, Jacob M, Cheatum CM 2017 Compressively sampled two-dimensional infrared spectroscopy that preserves line shape information. J. Phys. Chem. A 121, 3088–3093. (doi:10.1021/acs.jpca.7b01965) 28365984 10.1021/acs.jpca.7b01965

[103] Gundogdu K, Stone KW, Turner DB, Nelson KA 2007 Multidimensional coherent spectroscopy made easy. Chem. Phys. 341, 89–94. (doi:10.1016/j.chemphys.2007.06.027)

[104] Riedle E, Beutter M, Lochbrunner S, Piel J, Schenkl S, Spörlein S, Zinth W 2000 Generation of 10 to 50 fs pulses tunable through all of the visible and the NIR. Appl. Phys. B 71, 457–465. (doi:10.1007/s003400000351)

[105] Nisoli M, Danielius R, Piskarskas A, De Silvestri S, Magni V, Valiulis G, Varanavicius A, Svelto O 1994 Highly efficient parametric conversion of femtosecond Ti:sapphire laser pulses at 1 kHz. Opt. Lett. 19, 1973–1975. (doi:10.1364/OL.19.001973) 19855713 10.1364/ol.19.001973

[106] Zheng H, Caram JR, Dahlberg PD, Rolczynski BS, Viswanathan S, Dolzhnikov DS, Khadivi A, Talapin DV, Engel GS 2014 Dispersion-free continuum two-dimensional electronic spectrometer. Appl. Opt. 53, 1909 (doi:10.1364/AO.53.001909) 24663470 10.1364/AO.53.001909 PMC4349747

[107] Spokoyny B, Harel E 2014 Mapping the vibronic structure of a molecule by few-cycle continuum two-dimensional spectroscopy in a single pulse. J. Phys. Chem. Lett. 5, 2808–2814. (doi:10.1021/jz5012302) 26278083 10.1021/jz5012302

[108] Ma X, Dostál J, Brixner T 2016 Broadband 7-fs diffractive-optic-based 2D electronic spectroscopy using hollow-core fiber compression. Opt. Express 24, 20781 (doi:10.1364/OE.24.020781) 27607681 10.1364/OE.24.020781

[109] Kearns NM, Mehlenbacher RD, Jones AC, Zanni MT 2017 Broadband 2D electronic spectrometer using white light and pulse shaping: noise and signal evaluation at 1 and 100 kHz. Opt. Express 25, 7869 (doi:10.1364/OE.25.007869) 28380905 10.1364/OE.25.007869

[110] Durfee CG, Backus S, Kapteyn HC, Murnane MM 1999 Intense 8-fs pulse generation in the deep ultraviolet. Opt. Lett. 24, 697–699. (doi:10.1364/OL.24.000697) 18073827 10.1364/ol.24.000697

[111] Jailaubekov AE, Bradforth SE 2005 Tunable 30-femtosecond pulses across the deep ultraviolet. Appl. Phys. Lett. 87, 021107 (doi:10.1063/1.1992655)

[112] Varillas RB, Candeo A, Viola D, Garavelli M, De Silvestri S, Cerullo G, Manzoni C 2014 Microjoule-level, tunable sub-10 fs UV pulses by broadband sum-frequency generation. Opt. Lett. 39, 3849 (doi:10.1364/OL.39.003849) 24978753 10.1364/OL.39.003849

[113] Baum P, Lochbrunner S, Riedle E 2004 Generation of tunable 7-fs ultraviolet pulses: achromatic phase matching and chirp management. Appl. Phys. B 79, 1027–1032. (doi:10.1007/s00340-004-1668-2)

[114] Petersen PB, Tokmakoff A 2010 Source for ultrafast continuum infrared and terahertz radiation. Opt. Lett. 35, 1962–1964. (doi:10.1364/OL.35.001962) 20548353 10.1364/OL.35.001962

[115] Balasubramanian M, Courtney TL, Gaynor JD, Khalil M 2016 Compression of tunable broadband mid-IR pulses with a deformable mirror pulse shaper. J. Opt. Soc. Am. B 33, 2033–2035. (doi:10.1364/JOSAB.33.002033)

[116] Teo SM, Ofori-Okai BK, Werley CA, Nelson KA 2015 Invited article: single-shot THz detection techniques optimized for multidimensional THz spectroscopy. Rev. Sci. Instrum. 86, 051301 (doi:10.1063/1.4921389) 26026507 10.1063/1.4921389

[117] Elsaesser T, Reimann K, Woerner M 2015 Focus: phase-resolved nonlinear terahertz spectroscopy—from charge dynamics in solids to molecular excitations in liquids. J. Chem. Phys. 142, 212 301–212 310. (doi:10.1063/1.4916522) 10.1063/1.4916522 26049419

[118] Schlau-Cohen GS, De Re E, Cogdell RJ, Fleming GR 2012 Determination of excited-state energies and dynamics in the B band of the bacterial reaction center with 2D electronic spectroscopy. J. Phys. Chem. Lett. 3, 2487–2492. (doi:10.1021/jz300841u) 26292138 10.1021/jz300841u

[119] Scholes GD 2014 Extreme cross-peak 2D spectroscopy. Proc. Natl Acad. Sci. USA 111, 10 031–10 032. (doi:10.1073/pnas.1410105111) 10.1073/pnas.1410105111 PMC4104860 25002469

[120] Oliver TAA, Lewis NHC, Fleming GR 2014 Correlating the motion of electrons and nuclei with two-dimensional electronic-vibrational spectroscopy. Proc. Natl Acad. Sci. USA 111, 10 061–10 066. (doi:10.1073/pnas.1409207111) 10.1073/pnas.1409207111 PMC4104903 24927586

[121] Courtney TL, Fox ZW, Estergreen L, Khalil M 2015 Measuring coherently coupled intramolecular vibrational and charge-transfer dynamics with two-dimensional vibrational-electronic spectroscopy. J. Phys. Chem. Lett. 6, 1286–1292. (doi:10.1021/acs.jpclett.5b00356) 26262989 10.1021/acs.jpclett.5b00356

[122] Polli D et al. 2010 Conical intersection dynamics of the primary photoisomerization event in vision. Nature 467, 440–443. (doi:10.1038/nature09346) 20864998 10.1038/nature09346

[123] Johnson PJM, Halpin A, Morizumi T, Prokhorenko VI, Ernst OP, Miller RJD 2015 Local vibrational coherences drive the primary photochemistry of vision. Nat. Chem. 7, 980–986. (doi:10.1038/nchem.2398) 26587713 10.1038/nchem.2398

[124] Schreier WJ, Gilch P, Zinth W 2015 Early events of DNA photodamage. Annu. Rev. Phys. Chem. 66, 497–519. (doi:10.1146/annurev-physchem-040214-121821) 25664840 10.1146/annurev-physchem-040214-121821

[125] Kohler B 2010 Nonradiative decay mechanisms in DNA model systems. J. Phys. Chem. Lett. 1, 2047–2053. (doi:10.1021/jz100491x)

[126] Lewis NHC, Dong H, Oliver TAA, Fleming GR 2015 Measuring correlated electronic and vibrational spectral dynamics using line shapes in two-dimensional electronic-vibrational spectroscopy. J. Chem. Phys. 142, 174202 (doi:10.1063/1.4919686) 25956093 10.1063/1.4919686

[127] Dong H, Lewis NHC, Oliver TAA, Fleming GR 2015 Determining the static electronic and vibrational energy correlations via two-dimensional electronic-vibrational spectroscopy. J. Chem. Phys. 142, 174201 (doi:10.1063/1.4919684) 25956092 10.1063/1.4919684

[128] Oliver TAA, Fleming GR 2015 Following coupled electronic-nuclear motion through conical intersections in the ultrafast relaxation of β-Apo-8′-carotenal. J. Phys. Chem. B 119, 11 428–11 441. (doi:10.1021/acs.jpcb.5b04893) 10.1021/acs.jpcb.5b04893 26132534

[129] Lewis NHC, Dong H, Oliver TAA, Fleming GR 2015 A method for the direct measurement of electronic site populations in a molecular aggregate using two-dimensional electronic-vibrational spectroscopy. J. Chem. Phys. 143, 124203 (doi:10.1063/1.4931634) 26429003 10.1063/1.4931634

[130] Lewis NHC, Gruenke NL, Oliver TAA, Ballottari M, Bassi R, Fleming GR 2016 Observation of electronic excitation transfer through light harvesting complex II using two-dimensional electronic–vibrational spectroscopy. J. Phys. Chem. Lett. 7, 4197–4206. (doi:10.1021/acs.jpclett.6b02280) 10.1021/acs.jpclett.6b02280 PMC6314458 27704843

[131] Gaynor JD, Khalil M 2017 Signatures of vibronic coupling in two-dimensional electronic-vibrational and vibrational-electronic spectroscopies. J. Chem. Phys. 147, 094202 (doi:10.1063/1.4991745) 28886647 10.1063/1.4991745

[132] Mancal T, Christensson N, Lukeš V, Milota F, Bixner O, Kauffmann HF, Hauer J 2012 System-dependent signatures of electronic and vibrational coherences in electronic two-dimensional spectra. J. Phys. Chem. Lett. 3, 1497–1502. (doi:10.1021/jz300362k) 26285628 10.1021/jz300362k

[133] Nemeth A, Lukeš V, Sperling J, Milota F, Kauffmann HF, Mancal T 2009 Two-dimensional electronic spectra of an aggregating dye: simultaneous measurement of monomeric and dimeric line-shapes. Phys. Chem. Chem. Phys. 11, 5986–5997. (doi:10.1039/b902477h) 19588022 10.1039/b902477h

[134] Gaynor JD, Courtney TL, Balasubramanian M, Khalil M 2016 Fourier transform two-dimensional electronic-vibrational spectroscopy using an octave-spanning mid-IR probe. Opt. Lett. 41, 2895 (doi:10.1364/OL.41.002895) 27304316 10.1364/OL.41.002895

[135] Bode S, Quentmeier CC, Liao P-N, Hafi N, Barros T, Wilk L, Bittner F, Walla PJ 2009 On the regulation of photosynthesis by excitonic interactions between carotenoids and chlorophylls. Proc. Natl Acad. Sci. USA 106, 12 311–12 316. (doi:10.1073/pnas.0903536106) 19617542 10.1073/pnas.0903536106 PMC2714278

[136] Demmig-Adams B, Garab G, Adams WW III, Govindjee (eds). 2014 Non-photochemical quenching and energy dissipation in plants, algae and cyanobacteria, advances in photosynthesis and respiration. Berlin, Germany: Springer.

[137] Durchan M, Fuciman M, Slouf V, Keşan G, Polívka T 2012 Excited-state dynamics of monomeric and aggregated carotenoid 8'-Apo-β-carotenal. J. Phys. Chem. A 116, 12 330–12 338. (doi:10.1021/jp310140k) 10.1021/jp310140k 23176366

[138] Ragnoni E, Di Donato M, Iagatti A, Lapini A, Righini R 2015 Mechanism of the intramolecular charge transfer state formation in all-trans-β-apo-8'-carotenal: influence of solvent polarity and polarizability. J. Phys. Chem. B 119, 420–432. (doi:10.1021/jp5093288) 25495920 10.1021/jp5093288

[139] Domcke W, Yarkony DR, Köppel H (eds). 2004 Conical intersections: electronic structure, dynamics and spectroscopy. Singapore: World Scientific.

[140] Herzberg G, Longuet-Higgins HC 1963 Intersection of potential energy surfaces in polyatomic molecules. Discuss. Faraday Soc. 35, 77 (doi:10.1039/df9633500077)

[141] Christensson N, Milota F, Nemeth A, Sperling J, Kauffmann HF, Pullerits T, Hauer J 2009 Two-dimensional electronic spectroscopy of beta-carotene. J. Phys. Chem. B 113, 16 409–16 419. (doi:10.1021/jp906604j) 10.1021/jp906604j 19954155

[142] De Re E, Schlau-Cohen GS, Leverenz RL, Huxter VM, Oliver TAA, Mathies RA, Fleming GR 2014 Insights into the structural changes occurring upon photoconversion in the orange carotenoid protein from broadband two-dimensional electronic spectroscopy. J. Phys. Chem. B 118, 5382–5389. (doi:10.1021/jp502120h) 24779893 10.1021/jp502120h

[143] Di Donato M, Segado Centellas M, Lapini A, Lima M, Avila F, Santoro F, Cappelli C, Righini R 2014 Combination of transient 2D-IR experiments and ab initio computations sheds light on the formation of the charge-transfer state in photoexcited carbonyl carotenoids. J. Phys. Chem. B 118, 9613–9630. (doi:10.1021/jp505473j) 25050938 10.1021/jp505473j

[144] Duschinsky F 1937 On the interpretation of electronic spectra of polyatomic molecules. Acta Physicochimica URSS 7, 551–566.

[145] Pang Y, Prantil MA, Van Tassle AJ, Jones GA, Fleming GR 2009 Excited-state dynamics of 8'-apo-beta-caroten-8'-al and 7',7′-dicyano-7′-apo-beta-carotene studied by femtosecond time-resolved infrared spectroscopy. J. Phys. Chem. B 113, 13 086–13 095. (doi:10.1021/jp905758e) 10.1021/jp905758e 19736997

[146] Khalil M, Demirdöven N, Tokmakoff A 2003 Coherent 2D IR spectroscopy: molecular structure and dynamics in solution. J. Phys. Chem. A 107, 5258–5279. (doi:10.1021/jp0219247)

[147] Nakayama K, Nakano H, Hirao K 1998 Theoretical study of the π→ π* excited states of linear polyenes: the energy gap between 11Bu+ and 21Ag− states and their character. Int. J. Quantum Chem. 66, 157–175. (doi:10.1002/(sici)1097-461x(1998)66:2<157::aid-qua7>3.0.co;2-u)

[148] Scholes GD, Fleming GR, Olaya-Castro A, van Grondelle R 2011 Lessons from nature about solar light harvesting. Nat. Chem. 3, 763–774. (doi:10.1038/nchem.1145) 21941248 10.1038/nchem.1145

[149] Standfuss J, Terwisscha van Scheltinga AC, Lamborghini M, Kühlbrandt W 2005 Mechanisms of photoprotection and nonphotochemical quenching in pea light-harvesting complex at 2.5 A resolution. EMBO J. 24, 919–928. (doi:10.1038/sj.emboj.7600585) 15719016 10.1038/sj.emboj.7600585 PMC554132

[150] Schlau-Cohen GS, Calhoun TR, Ginsberg NS, Read EL, Ballottari M, Bassi R, van Grondelle R, Fleming GR 2009 Pathways of energy flow in LHCII from two-dimensional electronic spectroscopy. J. Phys. Chem. B 113, 15 352–15 363. (doi:10.1021/jp9066586) 19856954 10.1021/jp9066586

[151] Duan H-G, Stevens AL, Nalbach P, Thorwart M, Prokhorenko VI, Miller DRJ 2015 Two-dimensional electronic spectroscopy of light harvesting complex II at ambient temperature: a joint experimental and theoretical study. J. Phys. Chem. B 119, 12 017–12 027. (doi:10.1021/acs.jpcb.5b05592) 10.1021/acs.jpcb.5b05592 26301382

[152] Westenhoff S, Paleček D, Edlund P, Smith P, Zigmantas D 2012 Coherent picosecond exciton dynamics in a photosynthetic reaction center. J. Am. Chem. Soc. 134, 16 484–16 487. (doi:10.1021/ja3065478) 10.1021/ja3065478 23009768

[153] Ginsberg NS, Davis JA, Ballottari M, Cheng Y-C, Bassi R, Fleming GR 2011 Solving structure in the CP29 light harvesting complex with polarization-phased 2D electronic spectroscopy. Proc. Natl Acad. Sci USA 108, 3848–3853. (doi:10.1073/pnas.1012054108) 21321222 10.1073/pnas.1012054108 PMC3054007

[154] Reimers JR, Cai Z-L, Kobayashi R, Rätsep M, Freiberg A, Krausz E 2013 Assignment of the Q-bands of the chlorophylls: coherence loss via Qx − Qy mixing. Sci. Rep. 3, 1–8. (doi:10.1038/srep02761) 10.1038/srep02761 PMC3783888 24067303

[155] Jia Y, Jean JM, Werst MM, Chan C-K, Fleming GR 1992 Simulations of the temperature dependence of energy transfer in the PSI core antenna. Biophys. J. 63, 259–273. (doi:10.1016/S0006-3495(92)81589-8) 1420871 10.1016/S0006-3495(92)81589-8 PMC1262143

[156] Groot ML, Breton J, van Wilderen LJGW, Dekker JP, van Grondelle R 2004 Femtosecond visible/visible and visible/mid-IR pump–probe study of the photosystem II core antenna complex CP47. J. Phys. Chem. B 108, 8001–8006. (doi:10.1021/jp037966s)

[157] Groot ML, Pawlowicz NP, van Wilderen LJGW, Breton J, van Stokkum IHM, van Grondelle R 2005 Initial electron donor and acceptor in isolated photosystem II reaction centers identified with femtosecond mid-IR spectroscopy. Proc. Natl Acad. Sci. USA 102, 13 087–13 092. (doi:10.1073/pnas.0503483102) 10.1073/pnas.0503483102 PMC1196200 16135567

[158] Stahl AD, Di Donato M, van Stokkum I, van Grondelle R, Groot ML 2009 A femtosecond visible/visible and visible/mid-infrared transient absorption study of the light harvesting complex II. Biophys. J. 97, 3215–3223. (doi:10.1016/j.bpj.2009.09.037) 20006959 10.1016/j.bpj.2009.09.037 PMC2793356

[159] Di Donato M, Stahl AD, van Stokkum IHM, van Grondelle R, Groot ML 2011 Cofactors involved in light-driven charge separation in photosystem I identified by subpicosecond infrared spectroscopy. Biochemistry 50, 480–490. (doi:10.1021/bi101565w) 21155543 10.1021/bi101565w

[160] Zhu J, van Stokkum IHM, Paparelli L, Jones MR, Groot ML 2013 Early bacteriopheophytin reduction in charge separation in reaction centers of rhodobacter sphaeroides. Biophys. J. 104, 2493–2502. (doi:10.1016/j.bpj.2013.04.026) 23746522 10.1016/j.bpj.2013.04.026 PMC3672893

[161] Ohta K, Yang M, Fleming GR 2001 Ultrafast exciton dynamics of J-aggregates in room temperature solution studied by third-order nonlinear optical spectroscopy and numerical simulation based on exciton theory. J. Chem. Phys. 115, 7609–7621. (doi:10.1063/1.1403693)

[162] Lewis NHC, Fleming GR 2016 Two-dimensional electronic-vibrational spectroscopy of chlorophyll a and b. J. Phys. Chem. Lett. 7, 831–837. (doi:10.1021/acs.jpclett.6b00037) 26894783 10.1021/acs.jpclett.6b00037

[163] Cotton TM, Loach PA, Katz JJ, Ballschmitter K 1978 Studies of chlorophyll-chlorophyll and chlorophyll-ligand interactions by visible absorption and infrared spectroscopy at low temperature. Photochem. Photobiol. 27, 735–749. (doi:j.1751-1097.1978.tb07672.x)

[164] Novoderezhkin V, Marin A, van Grondelle R 2011 Intra- and inter-monomeric transfers in the light harvesting LHCII complex: the Redfield–Förster picture. Phys. Chem. Chem. Phys. 13, 17093 (doi:10.1039/c1cp21079c) 21866281 10.1039/c1cp21079c

[165] Ramanan C, Ferretti M, van Roon H, Novoderezhkin VI, van Grondelle R 2017 Evidence for coherent mixing of excited and charge-transfer states in the major plant light-harvesting antenna, LHCII. Phys. Chem. Chem. Phys. 19, 22 877–22 886. (doi:10.1039/C7CP03038J) 10.1039/c7cp03038j 28812075

[166] Courtney TL, Fox ZW, Slenkamp KM, Khalil M 2015 Two-dimensional vibrational-electronic spectroscopy. J. Chem. Phys. 143, 154201 (doi:10.1063/1.4932983) 26493900 10.1063/1.4932983

[167] Tekavec PF, Lott GA, Marcus AH 2007 Fluorescence-detected two-dimensional electronic coherence spectroscopy by acousto-optic phase modulation. J. Chem. Phys. 127, 214307 (doi:10.1063/1.2800560) 18067357 10.1063/1.2800560

[168] Draeger S, Roeding S, Brixner T 2017 Rapid-scan coherent 2D fluorescence spectroscopy. Opt. Express 25, 3259 (doi:10.1364/OE.25.003259) 28241542 10.1364/OE.25.003259

[169] Mastron JN, Tokmakoff A 2016 Two-photon-excited fluorescence-encoded infrared spectroscopy. J. Phys. Chem. A 120, 9178–9187. (doi:10.1021/acs.jpca.6b09158) 27802385 10.1021/acs.jpca.6b09158

[170] Lott GA, Perdomo-Ortiz A, Utterback JK, Widom JR, Aspuru-Guzik A, Marcus AH 2011 Conformation of self-assembled porphyrin dimers in liposome vesicles by phase-modulation 2D fluorescence spectroscopy. Proc. Natl Acad. Sci. USA 108, 16 521–16 526. (doi:10.1073/pnas.1017308108) 10.1073/pnas.1017308108 PMC3189026 21940499

[171] Perdomo-Ortiz A, Widom JR, Lott GA, Aspuru-Guzik A, Marcus AH 2012 Conformation and electronic population transfer in membrane-supported self-assembled porphyrin dimers by 2D fluorescence spectroscopy. J. Phys. Chem. B 116, 10 757–10 770. (doi:10.1021/jp305916x) 10.1021/jp305916x 22882118

[172] Widom JR, Johnson NP, Hippel von PH, Marcus AH 2013 Solution conformation of 2-aminopurine dinucleotide determined by ultraviolet two-dimensional fluorescence spectroscopy. New J. Phys. 15, 025028 (doi:10.1088/1367-2630/15/2/025028) 10.1088/1367-2630/15/2/025028 PMC3819147 24223491

[173] Nardin G, Autry TM, Silverman KL, Cundiff ST 2013 Multidimensional coherent photocurrent spectroscopy of a semiconductor nanostructure. Opt. Express 21, 28617 (doi:10.1364/OE.21.028617) 24514373 10.1364/OE.21.028617

[174] Vella E, Grégoire P, Li H, Tuladhar SM, Vezie M 2015 Two-dimensional coherent photocurrent excitation spectroscopy in a polymer solar cell. arXiv. (https://arxiv.org/abs/1506.07837)

[175] Karki KJ, Widom JR, Seibt J, Moody I, Lonergan MC, Pullerits T, Marcus AH 2014 1AD Coherent two-dimensional photocurrent spectroscopy in a PbS quantum dot photocell. Nat. Commun. 5, 5869 (doi:10.1038/ncomms6869) 25519819 10.1038/ncomms6869

[176] Xiong W, Laaser JE, Mehlenbacher RD, Zanni MT 2011 Adding a dimension to the infrared spectra of interfaces using heterodyne detected 2D sum-frequency generation (HD 2D SFG) spectroscopy. Proc. Natl Acad. Sci. USA 108, 20 902–20 907. (doi:10.1073/pnas.1115055108) 10.1073/pnas.1115055108 PMC3248474 22143772

[177] Laaser JE, Zanni MT 2013 Extracting structural information from the polarization dependence of one- and two-dimensional sum frequency generation spectra. J. Phys. Chem. A 117, 5875–5890. (doi:10.1021/jp307721y) 23140356 10.1021/jp307721y

[178] Ghosh A, Ho J-J, Serrano AL, Skoff DR, Zhang T, Zanni MT 2015 Two-dimensional sum-frequency generation (2D SFG) spectroscopy: summary of principles and its application to amyloid fiber monolayers. Faraday Discuss. 177, 493–505. (doi:10.1039/C4FD00173G) 25611039 10.1039/c4fd00173g PMC4398620

[179] Laaser JE, Skoff DR, Ho J-J, Joo Y, Serrano AL, Steinkruger JD, Gopalan P, Gellman SH, Zanni MT 2014 Two-dimensional sum-frequency generation reveals structure and dynamics of a surface-bound peptide. J. Am. Chem. Soc. 136, 956–962. (doi:10.1021/ja408682s) 24372101 10.1021/ja408682s PMC3956615

[180] Tokmakoff A, Fleming GR 1997 Two-dimensional Raman spectroscopy of the intermolecular modes of liquid CS2. J. Chem. Phys. 106, 2569–2582. (doi:10.1063/1.473361)

[181] Blank DA, Kaufman LJ, Fleming GR 1999 Fifth-order two-dimensional Raman spectra of CS_2_ are dominated by third-order cascades. J. Chem. Phys. 111, 3105 (doi:10.1063/1.479591)

[182] Kubarych KJ, Milne CJ, Lin S, Astinov V, Miller RJD 2002 Diffractive optics-based six-wave mixing: heterodyne detection of the full *χ*^(5)^ tensor of liquid CS_2_. J. Chem. Phys. 116, 2016–2042. (doi:10.1063/1.1429961)

[183] Kaufman LJ, Heo J, Ziegler LD, Fleming GR 2002 Heterodyne-detected fifth-order nonresonant Raman scattering from room temperature CS_2_. Phys. Rev. Lett. 88, 207402 (doi:10.1103/PhysRevLett.88.207402) 12005604 10.1103/PhysRevLett.88.207402

[184] Molesky BP, Giokas PG, Guo Z, Moran AM 2014 Multidimensional resonance Raman spectroscopy by six-wave mixing in the deep UV. J. Chem. Phys. 141, 114202 (doi:10.1063/1.4894846) 25240351 10.1063/1.4894846

[185] Molesky BP, Guo Z, Moran AM 2015 Femtosecond stimulated Raman spectroscopy by six-wave mixing. J. Chem. Phys. 142, 212405 (doi:10.1063/1.4914095) 26049425 10.1063/1.4914095

[186] Molesky BP, Guo Z, Cheshire TP, Moran AM 2016 Two-dimensional resonance Raman spectroscopy of oxygen- and water-ligated myoglobins. J. Chem. Phys. 145, 034203 (doi:10.1063/1.4958625) 27448880 10.1063/1.4958625

[187] Harel E 2017 Four-dimensional coherent electronic Raman spectroscopy. J. Chem. Phys. 146, 154201 (doi:10.1063/1.4979485) 28433038 10.1063/1.4979485

[188] Spencer AP, Hutson WO, Harel E 2017 Quantum coherence selective 2D Raman-2D electronic spectroscopy. Nat. Commun. 8, 14732 (doi:10.1038/ncomms14732) 28281541 10.1038/ncomms14732 PMC5353627

[189] Molesky BP, Guo Z, Cheshire TP 2016 Perspective: two-dimensional resonance Raman spectroscopy. J. Chem. Phys. 145, 180901 (doi:10.1063/1.4966194) 27846686 10.1063/1.4966194

[190] Guo Z, Molesky BP, Cheshire TP, Moran AM 2015 Elucidation of reactive wavepackets by two-dimensional resonance Raman spectroscopy. J. Chem. Phys. 143, 124202 (doi:10.1063/1.4931473) 26429002 10.1063/1.4931473

[191] Tiwari V, Peters WK, Jonas DM 2013 Electronic resonance with anticorrelated pigment vibrations drives photosynthetic energy transfer outside the adiabatic framework. Proc. Natl Acad. Sci. USA 110, 1203–1208. (doi:10.1073/pnas.1211157110) 23267114 10.1073/pnas.1211157110 PMC3557059

[192] Chenu A, Christensson N, Kauffmann HF, Mancal T 2013 Enhancement of vibronic and ground-state vibrational coherences in 2D spectra of photosynthetic complexes. Sci. Rep. 3, 2029 (doi:10.1038/srep02029) 23778355 10.1038/srep02029 PMC3693153

[193] Savolainen J, Ahmed S, Hamm P 2013 Two-dimensional Raman-terahertz spectroscopy of water. Proc. Natl Acad. Sci. USA 110, 20 402–20 407. (doi:10.1073/pnas.1317459110) 10.1073/pnas.1317459110 PMC3870715 24297930

[194] Hamm P, Shalit A 2017 Perspective: echoes in 2D-Raman-THz spectroscopy. J. Chem. Phys. 146, 130 901–130 911. (doi:10.1063/1.4979288) 10.1063/1.4979288 28390339

[195] Shalit A, Ahmed S, Savolainen J, Hamm P 2017 Terahertz echoes reveal the inhomogeneity of aqueous salt solutions. Nat. Chem. 9, 273–278. (doi:10.1038/nchem.2642) 28221356 10.1038/nchem.2642

[196] Finneran IA, Welsch R, Allodi MA, Miller TF III, Blake GA 2016 Coherent two-dimensional terahertz-terahertz-Raman spectroscopy. Proc. Natl Acad. Sci. USA 113, 6857–6861. (doi:10.1073/pnas.1605631113) 27274067 10.1073/pnas.1605631113 PMC4922159

[197] Kuehn W, Reimann K, Woerner M, Elsaesser T, Hey R 2011 Two-dimensional terahertz correlation spectra of electronic excitations in semiconductor quantum wells. J. Phys. Chem. B 115, 5448–5455. (doi:10.1021/jp1099046) 21171588 10.1021/jp1099046

[198] Woerner M, Kuehn W, Bowlan P 2013 Ultrafast two-dimensional terahertz spectroscopy of elementary excitations in solids. New J. Phys. 15, 065003 (doi:10.1088/1367-2630/15/6/065003)

[199] Lu J, Li X, Hwang HY, Ofori-Okai BK, Kurihara T, Suemoto T, Nelson KA 2017 Coherent two-dimensional terahertz magnetic resonance spectroscopy of collective spin waves. Phys. Rev. Lett. 118, 207204 (doi:10.1103/PhysRevLett.118.207204) 28581810 10.1103/PhysRevLett.118.207204

[200] Zhang Z, Wells KL, Seidel MT, Tan H-S 2013 Fifth-order three-dimensional electronic spectroscopy using a pump-probe configuration. J. Phys. Chem. B 117, 15 369–15 385. (doi:10.1021/jp4046403) 10.1021/jp4046403 23808641

[201] Mukamel S, Healion D, Zhang Y, Biggs JD 2013 Multidimensional attosecond resonant X-ray spectroscopy of molecules: lessons from the optical regime. Annu. Rev. Phys. Chem. 64, 101–127. (doi:10.1146/annurev-physchem-040412-110021) 23245522 10.1146/annurev-physchem-040412-110021 PMC3721744

[202] Kowalewski M, Bennett K, Dorfman KE, Mukamel S 2015 Catching conical intersections in the act: monitoring transient electronic coherences by attosecond stimulated X-ray Raman signals. Phys. Rev. Lett. 115, 193 003–193 006. (doi:10.1103/PhysRevLett.115.193003) 10.1103/PhysRevLett.115.193003 26588377

[203] Bennett K, Zhang Y, Kowalewski M, Hua W, Mukamel S 2016 Multidimensional resonant nonlinear spectroscopy with coherent broadband X-ray pulses. Phys. Scr. T169, 1–15. (doi:10.1088/0031-8949/T169/1/014002)

[204] Ashfold MNR, Murdock D, Oliver TAA 2017 Molecular photofragmentation dynamics in the gas and condensed phases. Annu. Rev. Phys. Chem. 68, 63–82. (doi:10.1146/annurev-physchem-052516-050756) 28142311 10.1146/annurev-physchem-052516-050756

[205] Fingerhut BP, Dorfman KE, Mukamel S 2013 Monitoring nonadiabatic dynamics of the RNA base uracil by UV pump–IR probe spectroscopy. J. Phys. Chem. Lett. 4, 1933–1942. (doi:10.1021/jz400776r) 23914288 10.1021/jz400776r PMC3728908

[206] Baum P, Lochbrunner S, Riedle E 2004 Tunable sub-10-fs ultraviolet pulses generated by achromatic frequency doubling. Opt. Lett. 29, 1686–1688. (doi:10.1364/OL.29.001686) 15309860 10.1364/ol.29.001686

[207] West BA, Womick JM, Moran AM 2011 Probing ultrafast dynamics in adenine with mid-UV four-wave mixing spectroscopies. J. Phys. Chem. A 115, 8630–8637. (doi:10.1021/jp204416m) 21756005 10.1021/jp204416m

[208] Tseng C-H, Sándor P, Kotur M, Weinacht TC, Matsika S 2012 Two-dimensional Fourier transform spectroscopy of adenine and uracil using shaped ultrafast laser pulses in the deep UV. J. Phys. Chem. A 116, 2654–2661. (doi:10.1021/jp207228b) 22074393 10.1021/jp207228b

[209] Auböck G, Consani C, van Mourik F, Chergui M 2012 Ultrabroadband femtosecond two-dimensional ultraviolet transient absorption. Opt. Lett. 37, 2337–2339. (doi:doi.org/10.1364/OL.37.002337) 22739900 10.1364/OL.37.002337

[210] Krebs N, Pugliesi I, Hauer J, Riedle E 2013 Two-dimensional Fourier transform spectroscopy in the ultraviolet with sub-20 fs pump pulses and 250–720 nm supercontinuum probe. New J. Phys. 15, 085016 (doi:10.1088/0034-4885/69/6/R06)

[211] Prokhorenko VI, Picchiotti A, Pola M, Dijkstra AG, Miller RJD 2016 New insights into the photophysics of DNA nucleobases. J. Phys. Chem. Lett. 7, 4445–4450. (doi:10.1021/acs.jpclett.6b02085) 27786479 10.1021/acs.jpclett.6b02085

[212] Baiz CR, Schach D, Tokmakoff A 2014 Ultrafast 2D IR microscopy. Opt. Express 22, 18 724–18 735. (doi:10.1364/OE.22.018724) 10.1364/OE.22.018724 PMC4162349 25089490

[213] Serrano AL, Ghosh A, Ostrander JS, Zanni MT 2015 Wide-field FTIR microscopy using mid-IR pulse shaping. Opt. Express 23, 17815 (doi:10.1364/OE.23.017815) 26191843 10.1364/OE.23.017815 PMC4813054

[214] Ostrander JS, Serrano AL, Ghosh A, Zanni MT 2016 Spatially resolved two-dimensional infrared spectroscopy via wide-field microscopy. ACS Photonics 3, 1315–1323. (doi:10.1021/acsphotonics.6b00297) 27517058 10.1021/acsphotonics.6b00297 PMC4976945

[215] Ohkita H et al. 2008 Charge carrier formation in polythiophene/fullerene blend films studied by transient absorption spectroscopy. J. Am. Chem. Soc. 130, 3030–3042. (doi:10.1021/ja076568q) 18278911 10.1021/ja076568q

[216] Grancini G, Polli D, Fazzi D, Cabanillas-Gonzalez J, Cerullo G, Lanzani G 2011 Transient absorption imaging of P3HT:PCBM photovoltaic blend: evidence for interfacial charge transfer state. J. Phys. Chem. Lett. 2, 1099–1105. (doi:10.1021/jz200389b)

[217] Liebel M, Kukura P 2013 Broad-band impulsive vibrational spectroscopy of excited electronic states in the time domain. J. Phys. Chem. Lett. 4, 1358–1364. (doi:10.1021/jz4004203) 26282153 10.1021/jz4004203

[218] Fischer MC, Wilson JW, Robles FE, Warren WS 2016 Invited review article: pump-probe microscopy. Rev. Sci. Instrum. 87, 031101 (doi:10.1063/1.4943211) 27036751 10.1063/1.4943211 PMC4798998

[219] Terada Y, Yoshida S, Takeuchi O, Shigekawa H 2010 Real-space imaging of transient carrier dynamics by nanoscale pump–probe microscopy. Nat. Photon. 4, 869–874. (doi:10.1038/nphoton.2010.235)

[220] Halpin A, Johnson PJM, Tempelaar R, Murphy RS, Knoester J, Jansen TLC, Miller RJD 2014 Two-dimensional spectroscopy of a molecular dimer unveils the effects of vibronic coupling on exciton coherences. Nat. Chem. 6, 196–201. (doi:10.1038/nchem.1834) 24557133 10.1038/nchem.1834

[221] Monahan DM, Whaley-Mayda L, Ishizaki A, Fleming GR 2015 Influence of weak vibrational-electronic couplings on 2D electronic spectra and inter-site coherence in weakly coupled photosynthetic complexes. J. Chem. Phys. 143, 065101 (doi:10.1063/1.4928068) 26277167 10.1063/1.4928068

[222] Holdaway DIH, Collini E, Olaya-Castro A 2016 Coherence specific signal detection via chiral pump-probe spectroscopy. J. Chem. Phys. 144, 194112 (doi:10.1063/1.4948943) 27208941 10.1063/1.4948943

[223] Holdaway DIH, Collini E, Olaya-Castro A 2017 Isolating the chiral contribution in optical two-dimensional chiral spectroscopy using linearly polarized light. Opt. Express 25, 6383 (doi:10.1364/OE.25.006383) 28380990 10.1364/OE.25.006383

[224] Fidler AF, Singh VP, Long PD, Dahlberg PD, Engel GS 2014 Dynamic localization of electronic excitation in photosynthetic complexes revealed with chiral two-dimensional spectroscopy. Nat. Commun. 5, 1–6. (doi:10.1038/ncomms4286) 10.1038/ncomms4286 PMC3976994 24504144

[225] Dutta B, Helbing J 2015 Optimized interferometric setup for chiral and achiral ultrafast IR spectroscopy. Opt. Express 23, 16449 (doi:10.1364/OE.23.016449) 26193616 10.1364/OE.23.016449

